# Effects of non-pharmacological interventions on youth with internet addiction: a systematic review and meta-analysis of randomized controlled trials

**DOI:** 10.3389/fpsyt.2023.1327200

**Published:** 2024-01-11

**Authors:** Yue-Shuai Jiang, Tian-Hong Liu, Dan Qin, Zi-Pu Wang, Xiao-Ya He, Yan-Nan Chen

**Affiliations:** ^1^School of Sports Management and Communication, Capital University of Physical Education and Sports, Beijing, China; ^2^Emilio Aguinaldo College, Manila, Philippines; ^3^Beijing Normal University, College of P.E. and Sports, Beijing, China

**Keywords:** non-pharmacological interventions, internet addiction, youth, meta-analysis, systematic review

## Abstract

**Objective:**

To assess the overall effectiveness of non-pharmacological interventions on internet addiction (IA) in youth.

**Method:**

Randomized controlled trials (RCTs) published from their inception to April 1, 2023 were searched in Cochrane, Embase, Medline, Web of Science, China National Knowledge Infrastructure, China Science and Technology Journal Database, Chinese BioMedical Literature Database, and WanFang Data. Two reviewers independently extracted data and evaluated bias using the Cochrane Risk of Bias tool.

**Results:**

Sixty-six studies performed from 2007 to 2023, with a total of 4,385 participants, were identified. The NPIs included group counseling, cognitive behavioral therapy, sports intervention, combined interventions, eHealth, educational intervention, positive psychology intervention, sand play intervention, and electrotherapy. The results revealed that NPIs significantly reduced IA levels (standardized mean difference, SMD: −2.01, 95% confidence interval, CI: −2.29 to −1.73, *I*^2^ = 93.0%), anxiety levels (SMD: −1.07, 95%CI: −1.41 to −0.73, *I*^2^ = 72.4%), depression levels (SMD: −1.11, 95%CI: −1.52 to −0.7, *I*^2^ = 84.3%), and SCL-90 (SMD: −0.75, 95%CI: −0.97 to −0.54, *I*^2^ = 27.7%). Subgroup analysis stratified by intervention measure showed that cognitive behavioral therapy, group counseling, sports intervention, combined intervention, educational intervention, positive psychology intervention, sandplay intervention, and mobile health were all effective in relieving symptoms of IA except electrotherapy.

**Conclusion:**

NPIs appear to be effective in the treatment of IA in youth, which would act as an alternative treatment of IA. Further studies with larger sample sizes and robust designs are needed.

## Introduction

Internet addiction (IA), a downside of rapidly developing digital technologies and growing Internet accessibility, has aroused global concern (1). It was initially defined by Young (1996) as “pathological and compulsive internet use” and its core psychopathology is impaired control (2). In 2018, the World Health Organization officially listed Internet Gaming Disorder, a disorder due to addictive behaviors and substantial use, in the 11th revision of the International Classification of Diseases (ICD-11) (3). Being immature physically, mentally and cognitively, the youth easily fall victim to IA. Currently, the global prevalence rate of IA has reached 7.02% (4), with adolescents and young adults in the majority, up to 25–30% of whom are being affected worldwide (5). In addition, since corona-virus disease 2019 (COVID-19), long isolation at home has witnessed an increase in sedentary behavior and screen time, which aggravated IA among youth (6). Typically featuring co-occurring disorders such as depression, obesity, and attention deficit disorder (7), IA can affect adolescents’ academic performance, reduce their quality of life, and even lead to social adjustment disorders. As a consequence, there is a desperate need for effective interventions against IA. Pharmacological and non-pharmacological interventions (NPIs) are the existing treatments for IA (8, 9). In contrast to NPIs, pharmacological treatments for IA, such as sertraline and buspirone, have limited efficacy and are likely to have side effects and complications (10, 11). Besides, severe adverse effects following drug administration, and drug resistance in long-term use cannot be avoided (12).

However, owing to their relatively higher safety profile and less side effects compared to pharmacological interventions (13), NPIs gradually arrest more attention from healthcare professionals. Described as a treatment without a registered medication, NPIs can be applied individually or in combination with other interventions (14). The main NPIs in use at present for IA include group counseling, cognitive behavioral therapy (CBT), sports intervention, combined interventions, eHealth, educational intervention, positive psychology intervention, sandplay intervention, and electrotherapy (15, 16). Widely as NPIs have been used for IA, the evidence of NPIs and their inconsistent effects found in numerous studies make it difficult to judge the overall efficacy of NPIs on IA (17–19). In a multicenter cluster randomized clinical trial (RCT), the use of CBT significantly eased symptoms of IA in adolescents (17). However, no statistically significant difference was identified in the results of another study using electrotherapy to treat IA (20). Limitations in previous studies also add to the necessity of more research, such as unclear classification of intervention methods. A systematic review of the effects of NPIs on IA conducted by Zajac (21) included only three types of interventions (CBT, family therapy, other approaches). Meanwhile, neither the therapeutic effects of psychological interventions on IA from a holistic perspective, nor the effects of positive psychology and exercise intervention on IA were explored in the study, which will increase the instability and contingency of the results. Besides, insufficient sample size is also a major drawback of previous literature. A research investigating the impact of psychological interventions on IA had only six included studies (16). Without sufficient data, the accuracy and objectivity of the results were compromised. Another limitation in previous reviews (22, 23) is that some only analyzed the impact of NPIs on IA based on a particular intervention method (such as psychological intervention) rather than involving various NPIs, resulting in a partial understanding of the effect of NPIs on IA. Finally, the datasets included in previous literature require updating, since quite a few studies on NPIs and IA have been published recently with new evidence and findings presented (17, 24). To sum up, the effect of NPIs on IA is unclear and needs further discussion. The aim of this review is, therefore, to determine the overall influence of NPIs on IA in youth by meta-analysis.

## Methods

Our meta-analysis was conducted following Cochrane Collaboration Handbook recommendations (25) and the PRISMA extension statement for systematic reviews incorporating pairwise meta-analyses (26). All analyses were performed based on previously published studies. Therefore, no ethical approval or patient consent was required. The PRISMA checklist could be seen in the Supplementary Appendix 1.

### Search strategies and study selection

An exhaustive strategic literature search was performed to identify relevant RCTs regarding the efficacy of NPIs on IA and other psychological outcomes in youth from the following databases: Web of Science, Medline, Embase, Cochrane, Chinese National Knowledge Infrastructure, Wanfang Data, China Science and Technology Journal Database, and Chinese Biomedical Literature Database from their inception to April 1, 2023. These studies were screened by using Boolean logic operators in combination with medical subject terms and keywords without any language and publication date restrictions. The following terms are used alone or jointly with each other: “IA disorder,” “internet gaming disorder,” “psychological intervention,” “sports intervention,” “tDCS,” “youth,” and “RCT.” A series of recursive searches were manually carried out as complementary retrieval from top journals (such as JAMA Psychiatry or Neuroscience and Biobehavioral Reviews) and major international conference proceedings to avoid suitable articles that meet our inclusion criterion (4, 27) being left out. Moreover, manual searches were also conducted on the references of NPIs reviews as well as articles that were presented in the form of abstracts. Details of search strategies in all databases were shown in Supplementary Appendix 2.

All citations were managed by Endnote X9 software (Thompson ISI Research Soft, Philadelphia, PA), and duplicate or overlapping publications were removed automatically. Titles and abstracts from the initial search were evaluated by 2 independent authors, and discrepancies in this progress were resolved by discussion or judged by a third author. Subsequently, a further full-text evaluation was performed to ensure the studies’ accuracy and integrity.

### Inclusion and exclusion criteria

The included studies must satisfy the following eligibility criteria.

### Populations

Participants ranged from 15 to 24 years old with IA, diagnosed by Young Diagnostic Questionnaire (YDQ) (28), Revised Chen Internet Addiction Scale (CIAS-R) (29), or other IA scales (30).

### Interventions

The interventions could be any NPIs (e.g., exercise, or CBT) (17, 31). No restrictions were set on the types, duration, or frequency of the interventions. Any form of pharmacological intervention was excluded (e.g., bupropion) (11).

### Comparators

The comparator group could be wait-list, usual care, or placebo groups.

### Outcomes

The primary outcome was IA, whose score was measured using a valid and reliable scale. Depression, anxiety, and other indicators for evaluating psychological symptoms were secondary outcome measures. If multiple scales were used to assess the same outcomes in one study, the main measurement of the outcomes was adopted for this review. For studies without specifying the primary outcome measure, the measurement obtained through the most commonly used scale was included.

### Study design

Only RCTs published without language restrictions were included as their information was more likely to be unbiased than other study designs.

### Data extraction and quality assessment

Two reviewers independently drew key information from the included articles. Information about the study design, first author, publication year, interventions, duration, IA, as well as the characteristics of participants such as age, sample size, and sex ratio were sorted into a pre-designed comprehensive Excel form. Disagreements between the two reviewers were resolved by discussion. For unreported essential data in the original studies, the authors would be contacted to retrieve the data.

Two independent reviewers assessed each publication’s risk of bias (ROB) using the Cochrane Risk of Bias tool. This instrument consists of seven items, and included studies were graded as having an uncertain, low, or high risk of bias, respectively (32). For selection bias, studies that clearly clarified the random sequence generation and allocation concealment method were assessed as having a low risk of bias; otherwise, the risk was regarded high. Performance and detection bias were evaluated primarily based on whether the participants, personnel, and result evaluators were blinded or not. For attrition bias, studies with essential data missing, particularly primary outcome data, were classified as high-risk, since incomplete data would directly affect our subsequent analysis. We evaluated selective bias based on whether or not the study lacked secondary outcomes or reported insufficient data, such as their features. For other potential bias, we categorized them based on the full-text search to find out the causes, whether due to less rigorous study designs or apparent inconsistency with past studies. The risk bias of all the above items would be judged as “unclear risk” if the study failed to address relevant items. In addition, the ROB aimed to assess the methodological quality but was not used as a criterion for study selection.

### Statistical analyses

Following the Cochrane Collaboration Handbook, a traditional pairwise meta-analysis was carried out using random effects models by STATA software version 14.0 (Stata, Inc., College Station, TX) (32). Firstly, *I*^2^ statistics were used to evaluate the heterogeneity of the studies. Low, moderate, and high heterogeneity were indicated by *I*^2^ values of 25, 50, and 75%, separately. Q statistical test was also performed, and *p* values less than 0.1 indicated substantial heterogeneity (33). Secondly, for continuous data, the standardized mean difference (SMD), defined as the absolute mean difference divided by the standard deviation (SD) or mean difference (MD), was calculated, along with the corresponding 95% confidence interval (CI). For dichotomous data, the effect size was calculated using the odds ratio with 95% CI to measure group effects. Thirdly, a comparison-adjusted funnel plot was constructed to determine the existence of publication bias by visually observing the plot’s asymmetry. The Egger test was carried out as a quantitative complement to the funnel plot in order to determine whether the *p* value was less than 0.05 (34). In order to investigate any variations or statistically significant distinctions between trials, a set of subgroup analyses were carried out. The following items were included in subgroup analyses: intervention duration (≥8 weeks and < 8 weeks), publication year (≥2015 vs. <2015), sample size (≥100 vs. <100), outcome measurement (e.g., YDQ vs. CIAS-R), region (China and non-China), population type (college students vs. primary and middle school students vs. others), intervention measure (CBT, group counseling, sports intervention, combined interventions, electrotherapy, educational intervention, positive psychology intervention, sandplay intervention, mobile health).

## Results

### Literature selection and characteristics of included studies

**Figure 1:**
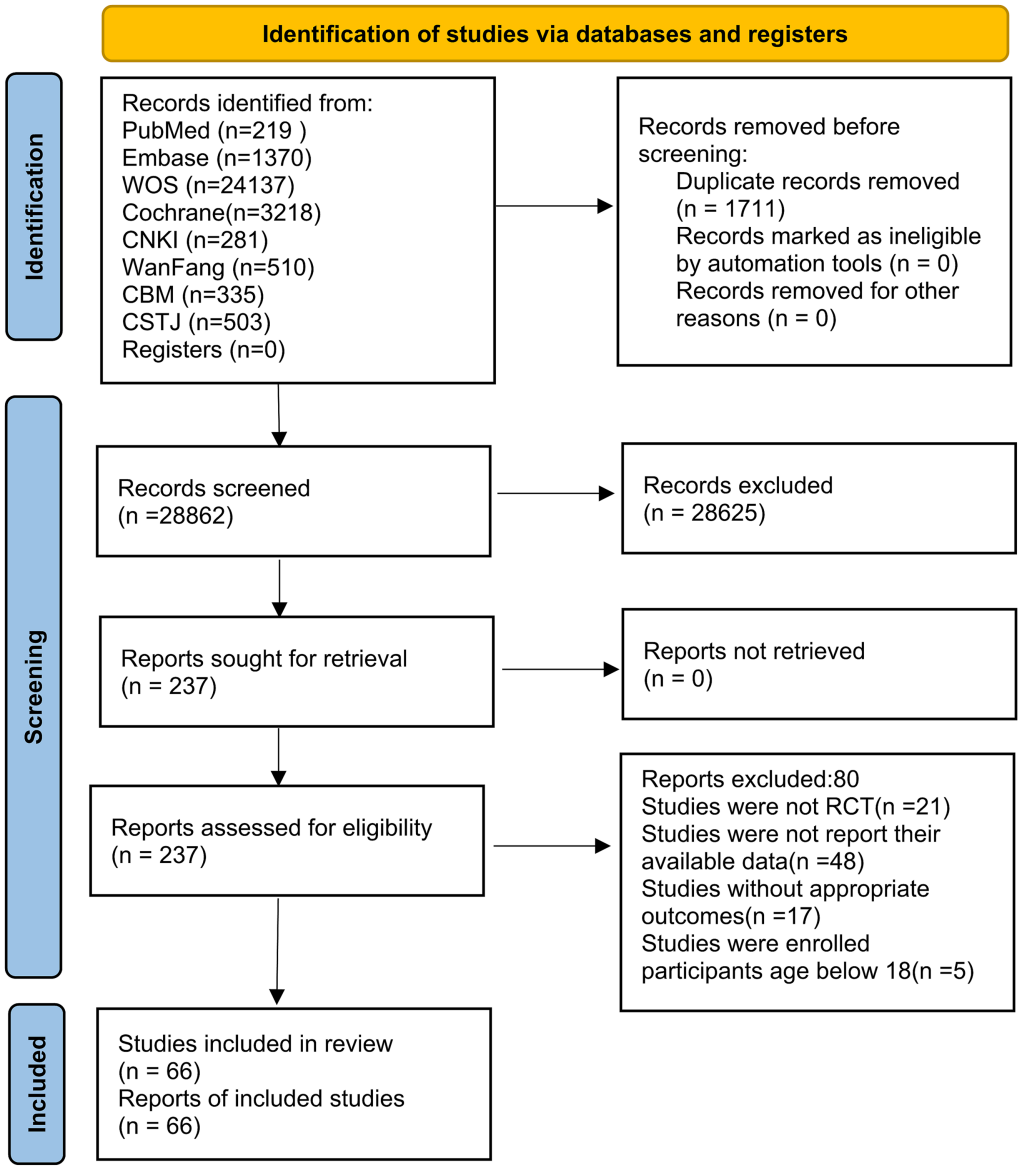
Literature review flowchart. CNKI, China National Knowledge Infrastructure; CMB, Chinese Biomedical; CSTJ, China Science and Technology Journal; WOS, Web of Science.

The initial literature search generated 29,299 records, and 437 duplicates were deleted,

leaving 28,862 publications for title and abstract review, after which only 237 remained for.

further screening. Eventually, 62 papers matched our requirements. To avoid omitting potentially relevant studies, the bibliographies of similar meta-analyses or reviews were examined, which ultimately identifying 4 additional studies that met our criteria. In total, 66 studies were included in our study (Supplementary Appendix 3). The article selection process is summarized in the PRISMA flow diagram in Figure 1. The search yielded a total of 66 trails from 7 nations (Israel, China, Korea, Turkey, Iran, Germany, Australia), with publication dates ranging from 2007 to 2022. In total, 2,150 participants were assigned to the IA group and 2,235 were assigned to the control group. All trial participants met at least one standard diagnostic criterion, such as Internet Addiction Test (IAT; *N* = 22), CIAS-R (*N* = 20), YDQ (*N* = 17), or other clinical diagnostic instruments (*N* = 7). The intervention duration for IA patients varied between 5 days and 20 weeks. Table 1 presents the characteristics of the included studies and the participants. Further description of demographic characteristics of the 66 trials is listed in Supplementary Table 1.

**Table 1:** Demographic characteristics of included studies.

Publication year		Intervention duration	
2005–2010	13 (20%)	≥8 weeks	41 (62%)
2011–2015	26 (39%)	<8 weeks	22 (33%)
2016–2020	18 (27%)	NR	3 (5%)
2021	9 (14%)	Geographic region	
Sample size		China	56 (85%)
≥100	11 (17%)	Others	10 (15%)
<100	55 (83%)	Diagnosis	
Intervention type		IAT	26 (39%)
CBT	16 (24%)	YDQ	13 (19%)
Group counselling	22 (33%)	CIAS-R	20 (30%)
Sports intervention	9 (14%)	K-IAS	1(2%)
Combined intervention	10 (15%)	CGAI	1(2%)
Electrotherapy	3 (4%)	PIUS	2(3%)
Educational intervention	2 (3%)	OGAS	1(2%)
Positive psychology intervention	2 (3%)	AICA-S	1(2%)
Sandplay intervention	1 (2%)	CIUS	1(2%)
Mobile health	1 (2%)		

AICA-S, Assessment of internet and computer game addiction self-report; CGAI, Computer game addiction inventory; CIUS, Compulsive internet use scale; CIAS-R, Revised chen internet addiction scale; CBT, Cognitive behavior therapy; IAT, Internet addiction test; K-IAS, Korea internet addiction scale; NR, Not reported; OGAS, Online game addiction scale; PIUS, Problematic internet use scale; YDQ, Young diagnostic questionnaire.

### Quality of included studies

Individual and overall study-level quality were plotted in Supplementary Figures 1, 2, respectively. All 66 included trials reported adequate random sequence generation, and 44 RCTs reported their strategy for allocation concealment. 57 RCTs had uncertain bias in terms of both performance and detection items, 1 had a high risk of performance bias and 5 had a high risk of detection bias. 65 RCTs were at low risk in terms of attrition bias. For additional bias items, 5 trials were deemed to have a high risk of bias.

### Primary outcome

#### Effects of NPIs on IA

All 66 trials investigated the effects of NPIs on IA between NPIs (2,150) and CG (control group; 2,235), and our results showed that NPIs were more effective than the CG in clinical practice with a statistical significance SMD of −2.01 (SMD = −2.01, 95%CI: −2.29 to −1.73, *I*^2^ = 93.0%, *P*_heterogeneity_ < 0.1; Table 2). The funnel plot was not symmetrical (Supplementary Figure 3), suggesting the existence of potential publication bias (*P*_egger_ < 0.05; Supplementary Figure 4).

**Table 2:** Primary results based on internet addiction and subgroup analyses.

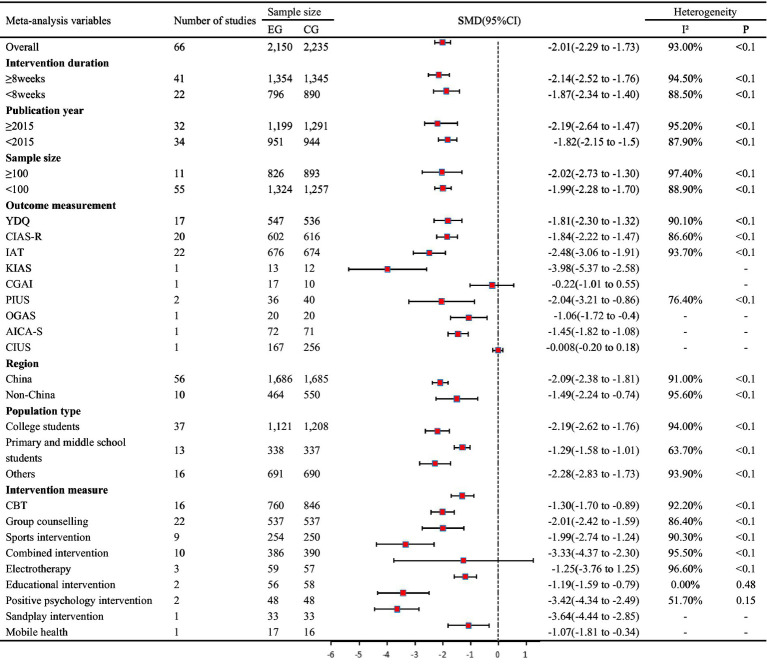

AICA-S, Assessment of internet and computer game addiction self-report; CBT, Cognitive behavior therapy; CIAS, Revised chen internet addiction scale; CGAI, Computer game addiction inventory; CIUS, Compulsive internet use scale; CG, Control group; CI, Confidence interval, Confidence interval; SMD, Standard mean differences; EG, Experimental group; IAT, Internet addiction test; KIAS, Korea internet addiction scale; OGAS, Online game addiction scale; PIUS, Problematic internet use scale; YDQ, Young diagnostic questionnaire.

### Secondary outcome

#### Depression

There were 11 studies focused on the effect of NPIs on depression (742 participants). The results showed that patients receiving NPIs significantly improved their depression compared to CG (SMD: −1.11, 95%CI: −1.52 to −0.70, *I*^2^ = 84.3%). The absence of symmetry in the funnel plot suggests the existence of publication bias (*P*_egger_ < 0.05; Supplementary Figure 5).

### Anxiety

Data from 11 studies reported the effects of NPIs on anxiety (615 participants). The outcome demonstrated that anxiety levels in NPIs groups was significantly lower than those in CG (SMD: −1.07, 95%CI −1.41 to −0.73, *I*^2^ = 72.4%). The asymmetry of the funnel plot for anxiety indicated publication bias (*P*_egger_ = 0.14; Supplementary Figure 6).

### Symptom Checklist-90

10 studies evaluated data on effects of NPIs on Symptom Checklist-90 (SCL-90; 528 participants). Pooled results showed that significant difference was found between the NPIs groups and CG (SMD: −0.75, 95%CI: −0.97 to −0.54, *I*^2^ = 27.7%). No symmetry was observed in the funnel plot, indicating publication bias (*P*_egger_ = 0.92; Supplementary Figure 7).

### Subgroup analyses

On the basis of the primary outcome of IA, subgroup analyses were performed with various factors of interest. The majority of analyses yielded consistency without statistically significant variations between subgroup items. Nevertheless, when the items of intervention measure and outcome measurement were taken into consideration, measurement of all interventions [e.g., CBT (SMD = −1.30, 95%CI: −1.70 to −0.89); sports intervention (SMD = −1.99, 95%CI: −2.74 to −1.24)] saw a notable improvement except that of the electrotherapy (SMD = −1.25, 95%CI = −3.76 to 1.25). The same result was also found for outcome measurement [e.g., YDQ (SMD = −1.81, 95%CI = −2.30 to −1.32)] compared with Computer Game Addiction Inventory (SMD = −0.22, 95%CI = −1.01 to 0.55) and Compulsive Internet Use Scale (SMD = −0.008, 95%CI = 0.20 to 0.18; Table 2). In addition, we also performed subgroup analysis on secondary outcome indicators, such as depression, anxiety, and SCL-90. The results are shown in Supplementary Tables 2–4, respectively.

## Discussion

From the overall results of our study involving 66 RCTs and 4,385 patients, compared with the control group, youth with IA receiving NPIs had more obvious alleviation in IA, depression, anxiety, and SCL-90.

The NPIs involved in the 66 studies varied considerably, so the types of NPIs were grouped into four categories: psychological intervention, sports intervention, electrotherapy, and combined intervention. Most of the NPIs included psychological intervention (CBT, group counseling, educational intervention, positive psychology intervention, sandplay intervention), and were found to improve the symptoms of IA (e.g., CBT, SMD = −1.30, 95%CI: −1.70 to −0.89). These results are consistent with previous findings concerning youth with a clinical diagnosis of IA (15, 17). A cluster RCT conducted a 12-month CBT intervention with 422 adolescents. The results showed that CBT significantly relieved IA symptoms in adolescents compared to the control group (17), which is consistent with the findings of a systematic review (35). Long-term online gaming can lead to decreased interpersonal relationships and increased loneliness (36), resulting in symptoms such as anxiety and depression, which exacerbated IA in turn (37, 38), causing a vicious cycle. Psychological intervention (such as CBT and positive psychological intervention) is thought to reduce IA level by improving the quality of interpersonal relationships, reducing loneliness, alleviating depression and anxiety (39).

NPIs based on sports intervention (aerobic exercise, Taichi, high-intensity interval training) had a large effect size on IA symptom (SMD = −1.99, 95%CI: −2.74 to −1.24), as a previous review concerning youth with IA showed (40, 41). Online games stimulate changes in the brain through the its reward system and also promote the release of dopamine and other neurotransmitters, increasing the pleasure of playing games in teenagers that gradually causes gaming dependency. As the process repeats, eventually there are changes in brain structure and addiction is formed (42, 43). By replacing online activity with sports intervention, the physiological and psychological well-being of the individual with IA is improved (43). For instance, exercise can weaken the addict’s desire for the Internet by promoting the secretion of dopamine and endophenolphthalein, which in turn reduces Internet dependence (19).

Electrotherapy was found to be ineffective in treating IA (SMD = −1.25, 95CI%: −3.76 to 1.25) in the pooled analysis. However, the results required further exploration since they were drawn from only three studies. Among the three studies, two used transcranial direct current stimulation as an intervention (18, 20), and one used repetitive transcranial magnetic stimulation (rTMS) as an intervention (44). The results showed that only rTMS was effective in the treatment of IA. The reason why addictive behaviors were suppressed may be that rTMS modulated the activity of neurotransmitters such as dopamine in the brain and acted on the left dorsolateral prefrontal cortex in humans (45). Brain imaging studies have also found that rTMS acting on the left dorsolateral prefrontal cortex of the brain can lead to inhibition of the cerebral cortex associated with addictive behavior or sensory seeking, thereby curbing an individual’s tendency for addictive behavior (46). Combinations of NPIs also produced improvements in IA (SMD = −3.33, 95%CI: −4.37 to −2.30), which is consistent with what has been found in recent studies (47, 48). In a RCT looking at combined interventions (high-intensity exercise plus nutritional intervention) among 80 students (47), subjects in the experimental group after 8 weeks have effectively suppressed their symptoms of IA compared to the control group. However, to determine whether combined NPIs are effective in preventing youth IA, more research is required as the evidence is relatively scant.

Another point worth emphasizing is that NPIs (CBT, exercise, combined intervention and group counseling) appeared to have positive effects on other psychological outcomes, including depression (SMD: −1.11, 95%CI: −1.52 to −0.70), anxiety (SMD: −1.07, 95%CI −1.41 to −0.73) and SCL-90 (SMD: −0.75, 95%CI: −0.97 to −0.54), which is consistent with previous research findings (49, 50). In our analysis, the most widely-used intervention to alleviate other psychological outcomes were CBT and group counseling. Focusing on releasing patients’ initiative and enthusiasm during the treatment process that is short in duration, CBT combines cognitive correction techniques with behavioral therapy techniques. Which is suitable for the treatment of various psychological disorders, such as depression (51) and anxiety (52). In addition, CBT enables patients to evaluate themselves correctly, help them reconstruct their cognitive structure and correct undesirable behaviors, and effectively reduce the pain of the cognitive revision process, thus enhancing their self-confidence and improving their psychological state (53). Group counseling interventions, which were found effective in improving mental health and alleviating symptoms of depression and anxiety (54, 55), are conducted in a group setting, with an emphasis on interpersonal interaction. In addition to attitudinal and behavioral change, group counseling techniques also stress emotional experience and self-awareness, introspection and self-growth. Group counseling can also be integrated with other psychological interventions (e.g., positive psychology) to enhance participants’ psychological well-being (56).

## Strengths and limitations

This meta-analysis has some advantages. First of all, this is the first meta-analysis to examine the holistic effect of NPIs on IA in youth, and the findings confirm that NPIs can significantly reduce IA in young people. Our research employed an exhaustive search strategy and multiple databases, as well as a complementary search for potential literature such as meetings and abstracts. As a result, the size of the study’s participant pool is substantial enough to support the estimated impacts statistically. Furthermore, NPIs can effectively avoid side effects of drugs, which is conducive to long-term sustained intervention and effective cessation of IA. As a consequence, this research may serve as a guide for decision-makers and clinicians in clinical decision-making, thereby benefiting future research and clinical application. Lastly, during the COVID-19, prolonged home isolation hindered access to drugs, and thus disrupted IA intervention experiments. In contrast, NPIs can be delivered through remote interventions such as Mobile Health (57) and will not be interrupted by COVID-19. Therefore, this study can provide a reference for sustained intervention for IA and sustained recovery of Internet addicts even in the face of major public crisis.

Several limitations should also be acknowledged. First, the inclusion of studies with varying IA diagnostic scales made it difficult to maintain homogeneity across studies. More objective and standardized IA diagnosis scale should be considered (e.g., YDQ). Second, the conclusion in this study should be extrapolated with care since a substantial proportion of the included studies were conducted in China, a country with a large population, where IA is a significant public health concern arousing intensive research attention. Based on this, we conducted a geographically-based subgroup analysis of studies from Chinese and non-Chinese regions. Third, the low quality of certain eligible studies may jeopardize the reliability of the findings, as some included studies did not perform blinding of participants or personnel, and were rated as having high-risk bias.

## Conclusion

The results showed that NPIs, in particular psychological intervention, sports and combined intervention, had significant effects on reducing IA in youth. To reach definitive findings, more studies with robust designs and sufficient sample sizes are required.

## Author contributions

Y-SJ: Conceptualization, Data curation, Methodology, Writing – original draft. T-HL: Supervision, Writing – review & editing. DQ: Formal analysis, Validation, Writing – review & editing. Z-PW: Conceptualization, Investigation, Software, Supervision, Writing – review & editing. X-YH: Data curation, Writing – review & editing. Y-NC: Software, Writing – review & editing.
