# Supplementary material for: Effects of non-pharmacological interventions on youth with internet addiction: a systematic review and meta-analysis of randomized controlled trials
Source: Front Psychiatry. 2024 Jan 11;14:1327200. doi: 10.3389/fpsyt.2023.1327200 (PMC10808612; doi:10.3389/fpsyt.2023.1327200)
Supplement: Supplementary file 1 [file Table_1.docx]

| **Supplementary Table 1 The Demographic Characteristics of Included Studies** | | | | | | | | |
| --- | --- | --- | --- | --- | --- | --- | --- | --- |
| Publication | Sample size | | Diagnose | Intervention type | | Intervention duration | Outcome | Region |
|  | EG | CG |  | EG | CG |  |  |  |
| Bai et al,2007 | 24 | 24 | CIAS-R | Group counselling | No intervention | 6weeks | ① | China |
| Kong et al,2011 | 36 | 35 | IAT | Group counselling | No intervention | 8weeks | ① | China |
| Cao et al,2007 | 29 | 35 | CIAS-R | CBT | Routine intervention | 9weeks | ① | China |
| Zheng et al,2007 | 30 | 31 | IAT | Group counselling | No intervention | 8weeks | ①④ | China |
| Kim,2008 | 13 | 12 | K-IAS | Group counselling | No intervention | 5weeks | ① | Republic of Korea |
| Guo et al,2008 | 16 | 16 | CIAS-R | Group counselling | Routine intervention | 1month | ②③ | China |
| Li et al,2009 | 38 | 38 | CIAS-R | CBT | No intervention | 8weeks | ① | China |
| Liao et al,2009 | 30 | 30 | CIAS-R | Sports intervention | No intervention | 10weeks | ① | China |
| Zhang,2009 | 35 | 35 | CIAS-R | Combined Interventions | No intervention | 12weeks | ①③ | China |
| Zhang et al,2009 | 12 | 13 | YDQ | Group counselling | No intervention | 8weeks | ① | China |
| Du et al,2010 | 32 | 24 | YDQ | CBT | No intervention | 9weeks | ①③ | China |
| Chen et al,2010 | 30 | 31 | IAT | Group counselling | No intervention | 8weeks | ①④ | China |
| Huang et al,2010 | 17 | 10 | CGAI | Group counselling | No intervention | 3weeks | ①②③ | China |
| Liu et al,2010 | 80 | 80 | IAT | Group counselling | Routine intervention | 4weeks | ① | China |
| Su et al,2011 | 17 | 16 | YDQ | eHealth | No intervention | 4weeks | ① | China |
| Chen et al,2011 | 14 | 23 | CIAS-R | Group counselling | No intervention | 7weeks | ①④ | China |
| Qiu et al,2011 | 18 | 18 | YDQ | Sports Intervention | No intervention | 12weeks | ① | China |
| Wang et al,2011 | 36 | 34 | CIAS-R | CBT | No intervention | 8weeks | ①④ | China |
| Ma et al,2011 | 49 | 49 | IAT | CBT | No intervention | NR | ① | China |
| Duan et al,2012 | 11 | 11 | IAT | Group counselling | No intervention | 3month | ① | China |
| Gao et al,2012 | 35 | 34 | IAT | Sports Intervention | No intervention | 8weeks | ①④ | China |
| Guan et al,2012 | 25 | 25 | CIAS-R | Group counselling | Routine intervention | 4weeks | ① | China |
| Lv et al,2012 | 26 | 26 | CIAS-R | Group counselling | Routine intervention | 8weeks | ① | China |
| Wang,2012 | 36 | 36 | IAT | CBT | Routine intervention | 3month | ① | China |
| Xu et al,2012 | 13 | 13 | YDQ | Group counselling | No intervention | 12weeks | ①②③ | China |
| Liu et al,2013 | 16 | 15 | IAT | Group counselling | Routine intervention | 13weeks | ① | China |
| Lu et al,2013 | 60 | 60 | IAT | Combined Interventions | Routine intervention | 5month | ① | China |
| Zhang ,2013 | 30 | 30 | IAT | Sports Intervention | No intervention | 4month | ① | China |
| Deng et al,2014 | 28 | 28 | YDQ | Combined Interventions | Routine intervention | 3month | ①④ | China |
| Ge et al,2014 | 12 | 12 | CIAS-R | Group counselling | No intervention | 5weeks | ① | China |
| Li et al,2014 | 27 | 24 | YDQ | Sports Intervention | No intervention | 10weeks | ① | China |
| Liu,2014 | 12 | 12 | IAT | CBT | No intervention | 8weeks | ① | China |
| Ren et al,2014 | 4 | 4 | YDQ | Sports Intervention | No intervention | 12weeks | ①④ | China |
| Wei et al,2014 | 60 | 60 | YDQ | Combined Interventions | Routine intervention | 20weeks | ①② | China |
| Liu et al,2015 | 21 | 25 | PIUS | Combined Interventions | Routine intervention | 18days | ① | China |
| Li et al,2015 | 42 | 42 | YDQ | Sports Intervention | No intervention | 16weeks | ① | China |
| Su,2015 | 15 | 15 | CIAS-R | Group counselling | No intervention | 6weeks | ① | China |
| Peng et al,2015 | 28 | 28 | YDQ | Group counselling | No intervention | 8weeks | ①④ | China |
| Zhao et al,2015 | 24 | 24 | CIAS-R | Group counselling | No intervention | 4weeks | ① | China |
| Cheng et al,2015 | 12 | 11 | IAT | Combined interventions | No intervention | 8weeks | ① | China |
| Çelik,2016 | 15 | 15 | PIUS | Educational intervention | No intervention | 5weeks | ① | Turkey |
| Fu et al,2016 | 42 | 42 | IAT | Sports Intervention | No intervention | 16weeks | ① | China |
| Li et al,2016 | 50 | 50 | IAT | Combined Interventions | Routine intervention | 3month | ①②③ | China |
| Su,2016 | 15 | 15 | CIAS-R | Group counselling | No intervention | 6weeks | ① | China |
| Zhao et al,2016 | 65 | 65 | CIAS-R | CBT | Routine intervention | 16weeks | ① | China |
| Khazaei et al,2017 | 24 | 24 | IAT | Positive psychology interventions | No intervention | 6weeks | ① | Iran |
| Li et al,2017 | 30 | 30 | YDQ | CBT | No intervention | 8weeks | ①②③④ | China |
| Yang et al,2017 | 26 | 26 | CIAS-R | Sports Intervention | No intervention | 16weeks | ① | China |
| Uysal et al,2018 | 41 | 43 | IAT | Educational intervention | Routine intervention | 3month | ① | Turkey |
| Chen et al,2018 | 52 | 52 | CIAS-R | CBT | Routine intervention | NR | ① | China |
| Xu et al,2018 | 20 | 20 | CIAS-R | Group counselling | Routine intervention | 1month | ①②③ | China |
| Zhang et al,2018 | 33 | 33 | IAT | Sandplay intervention | Routine intervention | 12weeks | ① | China |
| Wölfling et al,2019 | 72 | 71 | AICA-S | CBT | No intervention | 15weeks | ①② | Germany and Austria |
| Fang et al,2019 | 24 | 24 | IAT | Positive psychology interventions | No intervention | 8weeks | ① | China |
| Jeong et al,2020 | 13 | 13 | IAT | Electrotherapy | sham tDCS | 4weeks | ① | Republic of Korea |
| Zhong,2020 | 32 | 32 | IAT | Electrotherapy | sham tDCS | 4weeks | ① | China |
| Wen et al,2020 | 40 | 40 | CIAS-R | Combined Interventions | No intervention | 8weeks | ① | China |
| Agbaria,2021 | 80 | 80 | IAT | CBT | Routine intervention | 12weeks | ① | Israel |
| Lee et al,2021 | 14 | 12 | IAT | Electrotherapy | sham tDCS | 5days | ① | Republic of Korea |
| Alavi et al,2021 | 25 | 25 | IAT | CBT | Routine intervention | 10weeks | ①④ | Iran |
| Liu et al,2021 | 60 | 61 | CIAS-R | Combined Interventions | Routine intervention | 8times | ①②③ | China |
| Lu et al,2021 | 16 | 17 | IAT | CBT | No intervention | 5weeks | ①②③ | China |
| Gong et al,2022 | 60 | 58 | YDQ | Group counselling | No intervention | 8weeks | ① | China |
| Yang et al,2022 | 21 | 22 | IAT | CBT | No intervention | 6weeks | ① | China |
| Yang et al,2022 | 20 | 20 | OGAS | Combined interventions | No intervention | 15days | ①②③ | China |
| Lindenberg et al,2022 | 167 | 256 | CIUS | CBT | No intervention | 4session | ① | Germany |
| AICA-S, Assessment of internet and computer game addiction self-report; CIAS-R, Revised chen internet addiction scale; CG, Control group; CBT, Cognitive behavior therapy; CGAI, Computer game addiction inventory; CIUS, Compulsive internet use scale; EG, Experimental group; IAT, Internet addiction test; K-IAS, Korea internet addiction scale; NR, Not reported; OGAS, Online game addiction scale; PIUS, Problematic internet use scale; tDCS, Transcranial direct current stimulation; YDQ, Young diagnostic questionnaire; ①, Internet addiction; ②, Depression; ③, Anxiety; ④, SCL-90. | | | | | | | | |
