# Supplementary material for: Effects of non-pharmacological interventions on youth with internet addiction: a systematic review and meta-analysis of randomized controlled trials
Source: Front Psychiatry. 2024 Jan 11;14:1327200. doi: 10.3389/fpsyt.2023.1327200 (PMC10808612; doi:10.3389/fpsyt.2023.1327200)
Supplement: Supplementary file 2 [file Table_2.docx]

| **Table 2 Primary results based on internet addiction and subgroup analyses** | | | | | | |
| --- | --- | --- | --- | --- | --- | --- |
| Meta-analysis variables | Number of studies | Sample size | | SMD(95%CI) | Heterogeneity | |
|  |  | EG | CG |  | I² | P |
| Overall | 66 | 2,150 | 2,235 | -2.01(-2.29 to -1.73) | 93.00% | <0.1 |
| **Intervention duration** |  |  |  |  |  |  |
| ≥8weeks | 41 | 1,354 | 1,345 | -2.14(-2.52 to -1.76) | 94.50% | <0.1 |
| <8weeks | 22 | 796 | 890 | -1.87(-2.34 to -1.40) | 88.50% | <0.1 |
| **Publication year** |  |  |  |  |  |  |
| ≥2015 | 32 | 1,199 | 1,291 | -2.19(-2.64 to -1.47) | 95.20% | <0.1 |
| <2015 | 34 | 951 | 944 | -1.82(-2.15 to -1.5) | 87.90% | <0.1 |
| **Sample size** |  |  |  | 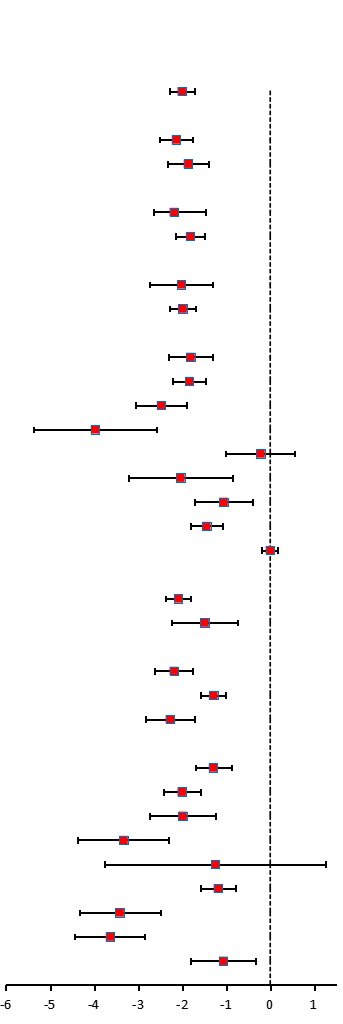 |  |  |
| ≥100 | 11 | 826 | 893 | -2.02(-2.73 to -1.30) | 97.40% | <0.1 |
| <100 | 55 | 1,324 | 1,257 | -1.99(-2.28 to -1.70) | 88.90% | <0.1 |
| **Outcome measurement** |  |  |  |  |  |  |
| YDQ | 17 | 547 | 536 | -1.81(-2.30 to -1.32) | 90.10% | <0.1 |
| CIAS-R | 20 | 602 | 616 | -1.84(-2.22 to -1.47) | 86.60% | <0.1 |
| IAT | 22 | 676 | 674 | -2.48(-3.06 to -1.91) | 93.70% | <0.1 |
| KIAS | 1 | 13 | 12 | -3.98(-5.37 to -2.58) |  | - |
| CGAI | 1 | 17 | 10 | -0.22(-1.01 to 0.55) |  | - |
| PIUS | 2 | 36 | 40 | -2.04(-3.21 to -0.86) | 76.40% | <0.1 |
| OGAS | 1 | 20 | 20 | -1.06(-1.72 to -0.4) | - | - |
| AICA-S | 1 | 72 | 71 | -1.45(-1.82 to -1.08) | - | - |
| CIUS | 1 | 167 | 256 | -0.008(-0.20 to 0.18) | - | - |
| **Region** |  |  |  |  |  |  |
| China | 56 | 1,686 | 1,685 | -2.09(-2.38 to -1.81) | 91.00% | <0.1 |
| Non-China | 10 | 464 | 550 | -1.49(-2.24 to -0.74) | 95.60% | <0.1 |
| **Population type** |  |  |  |  |  |  |
| College students | 37 | 1,121 | 1,208 | -2.19(-2.62 to -1.76) | 94.00% | <0.1 |
| Primary and middle school students | 13 | 338 | 337 | -1.29(-1.58 to -1.01) | 63.70% | <0.1 |
| Others | 16 | 691 | 690 | -2.28(-2.83 to -1.73) | 93.90% | <0.1 |
| **Intervention measure** |  |  |  |  |  |  |
| CBT | 16 | 760 | 846 | -1.30(-1.70 to -0.89) | 92.20% | <0.1 |
| Group counselling | 22 | 537 | 537 | -2.01(-2.42 to -1.59) | 86.40% | <0.1 |
| Sports intervention | 9 | 254 | 250 | -1.99(-2.74 to -1.24) | 90.30% | <0.1 |
| Combined intervention | 10 | 386 | 390 | -3.33(-4.37 to -2.30) | 95.50% | <0.1 |
| Electrotherapy | 3 | 59 | 57 | -1.25(-3.76 to 1.25) | 96.60% | <0.1 |
| Educational intervention | 2 | 56 | 58 | -1.19(-1.59 to -0.79) | 0.00% | 0.48 |
| Positive psychology intervention | 2 | 48 | 48 | -3.42(-4.34 to -2.49) | 51.70% | 0.15 |
| Sandplay intervention | 1 | 33 | 33 | -3.64(-4.44 to -2.85) | - | - |
| Mobile health | 1 | 17 | 16 | -1.07(-1.81 to -0.34) | - | - |
| AICA-S, Assessment of internet and computer game addiction self-report; CBT, Cognitive behavior therapy; CIAS, Revised chen internet addiction scale; CGAI, Computer game addiction inventory; CIUS, Compulsive internet use scale; CG, Control group; CI, Confidence interval, Confidence interval; SMD, Standard mean differences; EG, Experimental group; IAT, Internet addiction test; KIAS, Korea internet addiction scale; OGAS, Online game addiction scale; PIUS, Problematic internet use scale; YDQ, Young diagnostic questionnaire. | | | | | | |
