# Supplementary material for: Effects of non-pharmacological interventions on youth with internet addiction: a systematic review and meta-analysis of randomized controlled trials
Source: Front Psychiatry. 2024 Jan 11;14:1327200. doi: 10.3389/fpsyt.2023.1327200 (PMC10808612; doi:10.3389/fpsyt.2023.1327200)
Supplement: Supplementary file 3 [file Table_3.docx]

| **Supplementary Table 3 Primary results based on anxiety and subgroup analyses** | | | | | | |
| --- | --- | --- | --- | --- | --- | --- |
| Meta-analysis variables | Number of studies | Sample size | | SMD(95%CI) | Heterogeneity | |
|  |  | EG | CG |  | I² | P |
| Overall | 11 | 309 | 306 | -1.07(-1.41 to -0.73) | 72.4% | <0.1 |
| **Intervention duration** |  |  |  |  |  |  |
| ≥8weeks | 5 | 160 | 162 | -1.09(-1.67 to -0.52) | 81.7% | <0.1 |
| <8weeks | 5 | 149 | 144 | -1.23(-1.56 to -0.9) | 0.0% | 0.89 |
| **Publication year** |  |  |  |  |  |  |
| ≥2015 | 6 | 196 | 198 | 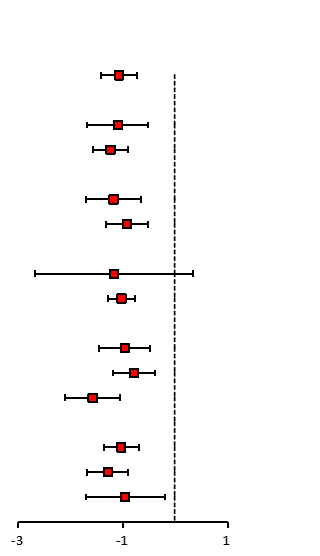-1.17(-1.69 to -0.64) | 81.7% | <0.1 |
| <2015 | 5 | 110 | 108 | -0.92(-1.31 to -0.52) | 45.7% | 0.11 |
| **Sample size** |  |  |  |  |  |  |
| ≥100 | 2 | 110 | 111 | -1.16(-2.67 to 0.35) | 96.1% | <0.1 |
| <100 | 9 | 199 | 195 | -1.02(-1.27 to -0.77) | 25.0% | 0.22 |
| **Population type** |  |  |  |  |  |  |
| College students | 4 | 125 | 128 | -0.96(-1.45 to -0.48) | 57.6% | <0.1 |
| Primary and middle school students | 4 | 98 | 92 | -0.78(-1.19 to -0.38) | 51.6% | 0.1 |
| Others | 3 | 86 | 86 | -1.57(-2.09 to -1.05) | 50.7% | 0.13 |
| **Intervention measure** |  |  |  |  |  |  |
| CBT | 3 | 78 | 81 | -1.03(-1.36 to -0.69) | 0.0% | 0.4 |
| Group counselling | 4 | 66 | 59 | -1.28(-1.67 to -0.89) | 0.0% | 0.83 |
| Combined interventions | 4 | 165 | 166 | -0.95(-1.70 to -0.20) | 90.0% | <0.1 |
| CBT, Cognitive behavior therapy; CG, Control group; CI, Confidence interval; SMD, Standard mean differences; EG, Experimental group. | | | | | | |
