# Supplementary material for: Effects of non-pharmacological interventions on youth with internet addiction: a systematic review and meta-analysis of randomized controlled trials
Source: Front Psychiatry. 2024 Jan 11;14:1327200. doi: 10.3389/fpsyt.2023.1327200 (PMC10808612; doi:10.3389/fpsyt.2023.1327200)
Supplement: Supplementary file 4 [file Table_4.docx]

| **Supplementary Table 4 Primary results based on SCL-90 and subgroup analyses** | | | | | | |
| --- | --- | --- | --- | --- | --- | --- |
| Meta-analysis variables | Number of studies | Sample size | | SMD(95%CI) | Heterogeneity | |
|  |  | EG | CG |  | I² | P |
| Overall | 10 | 260 | 268 | -0.75(-0.97 to -0.54) | 27.7% | 0.18 |
| **Intervention duration** |  |  |  |  |  |  |
| ≥8weeks | 9 | 246 | 235 | -0.76(-1.00 to -0.53) | 35.0% | 0.13 |
| <8weeks | 1 | 14 | 23 | -0.63(-1.31 to 0.04) | - | - |
| **Publication year** |  |  |  |  |  |  |
| ≥2015 | 3 | 83 | 83 | -0.73(-1.29 to -0.17) | 67.5% | <0.1 |
| <2015 | 7 | 177 | 185 | -0.77(-0.99 to -0.54) | 4.2% | 0.39 |
| **Region** |  |  |  |  |  |  |
| China | 9 | 235 | 243 | -0.81(-1.01 to -0.61) | 13.4% | 0.32 |
| Non-China | 1 | 25 | 25 | -0.27(-0.83 to 0.27) | - | - |
| **Population type** |  |  |  |  |  |  |
| College students | 7 | 168 | 176 | 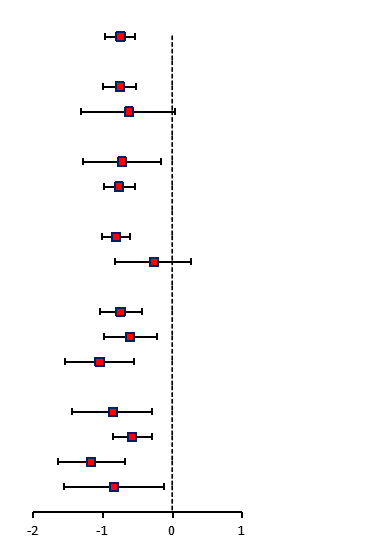-0.75(-1.05 to -0.44) | 43.0% | 0.1 |
| Primary and middle school students | 2 | 56 | 56 | -0.61(-0.99 to -0.23) | 0.0% | 0.81 |
| Others | 1 | 36 | 34 | -1.05(-1.55 to -0.55) | - | - |
| **Intervention measure** |  |  |  |  |  |  |
| CBT | 3 | 91 | 89 | -0.86(-1.44 to -0.29) | 70.7% | <0.1 |
| Group counselling | 4 | 102 | 113 | -0.58(-0.85 to -0.30) | 0.0% | 0.97 |
| Sports | 2 | 39 | 38 | -1.17(-1.65 to -0.68) | 0.0% | 0.85 |
| Combined interventions | 1 | 28 | 28 | -0.84(-1.56 to -0.12) | - | - |
| CBT, Cognitive behavior therapy; CG, Control group; CI, Confidence interval; SMD, Standard mean differences; EG, Experimental group; SCL, Symptom checklist. | | | | | | |
