# Supplementary material for: Effects of non-pharmacological interventions on youth with internet addiction: a systematic review and meta-analysis of randomized controlled trials
Source: Front Psychiatry. 2024 Jan 11;14:1327200. doi: 10.3389/fpsyt.2023.1327200 (PMC10808612; doi:10.3389/fpsyt.2023.1327200)
Supplement: Supplementary file 6 [file Data_Sheet_2.docx]

**Web of science Search Strategy**

1: (((((((((((((((((((((((((((((((((((((((((((((((((TS=(Internet Addiction Disorder)) OR TS=(Addiction Disorder, Internet)) OR TS=(Addiction Disorders, Internet)) OR TS=(Disorder, Internet Addiction)) OR TS=(Disorders, Internet Addiction)) OR TS=(Internet Addiction Disorders)) OR TS=(Internet Addiction)) OR TS=(Addiction, Internet)) OR TS=(Addictions, Internet)) OR TS=(Internet Addictions)) OR TS=(Social Media Addiction)) OR TS=(Addiction, Social Media)) OR TS=(Addictions, Social Media)) OR TS=(Media Addiction, Social)) OR TS=(Media Addictions, Social)) OR TS=(Social Media Addictions)) OR TS=(Smartphone Addiction)) OR TS=(Addiction, Smartphone)) OR TS=(Addictions, Smartphone)) OR TS=(Smartphone Addictions)) OR TS=(Internet Gaming Disorder)) OR TS=(Disorder, Internet Gaming)) OR TS=(Disorders, Internet Gaming)) OR TS=(Gaming Disorder, Internet)) OR TS=(Gaming Disorders, Internet)) OR TS=(Internet Gaming Disorders)) OR TS=(problematic Internet use)) OR TS=(internet use disorder)) OR TS=(internet use)) OR TS=(Internet Uses)) OR TS=(Use, Internet)) OR TS=(Web Usage)) OR TS=(Usage, Web)) OR TS=(Web Use)) OR TS=(Use, Web)) OR TS=(Internet Usage)) OR TS=(Usage, Internet)) OR TS=(Internet-addicted)) OR TS=(Internet depend)) OR TS=(Internet overuse)) OR TS=(Compulsive internet)) OR TS=(Pathological internet)) OR TS=(Excessive internet)) OR TS=(Addictive internet)) OR TS=(Video game addiction)) OR TS=(Online game addiction)) OR TS=(Cybersex addiction)) OR TS=(Internet sex addiction)) OR TS=(Social network addiction)) OR TS=(Facebook addiction) Date of operation: Sat Apr 1 2023 14:53:31 GMT+0800 (China Standard Time) Search Results: 656742

2: TS= clinical trial* OR TS=research design OR TS=comparative stud* OR TS=evaluation stud* OR TS=controlled trial* OR TS=follow-up stud* OR TS=prospective stud* OR TS=random* OR TS=placebo* OR TS=(single blind*) OR TS=(double blind*) Date of operation: Sat Apr 1 2023 14:56:29 GMT+0800 (China Standard Time) Search Results: 12754470

3: (((((((((((((((((((((((((((((TS=(Behavioral Therapies, Cognitive)) OR TS=(Cognitive Behavioral Therapy)) OR TS=(Behavioral Therapy, Cognitive)) OR TS=(Cognitive Behavioral Therapies)) OR TS=(Therapies, Cognitive Behavioral)) OR TS=(Therapy, Cognitive Behavioral)) OR TS=(Psychotherapy, Cognitive)) OR TS=(Therapy, Cognitive)) OR TS=(Cognitive Therapies)) OR TS=(Therapies, Cognitive)) OR TS=(Cognitive Therapy)) OR TS=(Cognitive Behaviour Therapy)) OR TS=(Behaviour Therapies, Cognitive)) OR TS=(Behaviour Therapy, Cognitive)) OR TS=(Cognitive Behaviour Therapies)) OR TS=(Therapies, Cognitive Behaviour)) OR TS=(Therapy, Cognitive Behaviour)) OR TS=(Cognitive Psychotherapy)) OR TS=(Cognitive Psychotherapies)) OR TS=(Psychotherapies, Cognitive)) OR TS=(Cognition Therapy)) OR TS=(Cognition Therapies)) OR TS=(Therapies, Cognition)) OR TS=(Therapy, Cognitive Behavior)) OR TS=(Behavior Therapies, Cognitive)) OR TS=(Cognitive Behavior Therapies)) OR TS=(Therapies, Cognitive Behavior)) OR TS=(Therapy, Cognition)) OR TS=(Behavior Therapy, Cognitive)) OR TS=(Cognitive Behavior Therapy) Date of operation: Sat Apr 1 2023 14:59:44 GMT+0800 (China Standard Time) Search Results: 340553

4: ((((((((((((((((((((((((((((((((((((((((TS=(Exercises)) OR TS=(exercise)) OR TS=(Physical Activity)) OR TS=(Activities, Physical)) OR TS=(Activity, Physical)) OR TS=(Physical Activities)) OR TS=(Exercise, Physical)) OR TS=(Exercises, Physical)) OR TS=(Physical Exercise)) OR TS=(Physical Exercises)) OR TS=(Acute Exercise)) OR TS=(Acute Exercises)) OR TS=(Exercise, Acute)) OR TS=(Exercises, Acute)) OR TS=(Exercise, Isometric)) OR TS=(Exercises, Isometric)) OR TS=(Isometric Exercises)) OR TS=(Isometric Exercise)) OR TS=(Exercise, Aerobic)) OR TS=(Aerobic Exercise)) OR TS=(Aerobic Exercises)) OR TS=(Exercises, Aerobic)) OR TS=(Exercise Training)) OR TS=(Exercise Trainings)) OR TS=(Training, Exercise)) OR TS=(Trainings, Exercise)) OR TS=(football)) OR TS=(Tai Chi)) OR TS=(basketball)) OR TS=(High-Intensity Interval Training)) OR TS=(High Intensity Interval Training)) OR TS=(High-Intensity Interval Trainings)) OR TS=(Interval Training, High-Intensity)) OR TS=(Interval Trainings, High-Intensity)) OR TS=(Training, High-Intensity Interval)) OR TS=(Trainings, High-Intensity Interval)) OR TS=(High-Intensity Intermittent Exercise)) OR TS=(Exercise, High-Intensity Intermittent)) OR TS=(High-Intensity Intermittent Exercises)) OR TS=(Sprint Interval Training)) OR TS=(Sprint Interval Trainings) Date of operation: Sat Apr 1 2023 15:04:36 GMT+0800 (China Standard Time) Search Results: 1620272

5: (((((((((((((((((((((((TS=(tDCS)) OR TS=(Cathodal Stimulation Transcranial Direct Current Stimulation)) OR TS=(Cathodal Stimulation tDCS)) OR TS=(Cathodal Stimulation tDCSs)) OR TS=(Stimulation tDCS, Cathodal)) OR TS=(Stimulation tDCSs, Cathodal)) OR TS=(tDCS, Cathodal Stimulation)) OR TS=(tDCSs, Cathodal Stimulation)) OR TS=(Transcranial Random Noise Stimulation)) OR TS=(Transcranial Alternating Current Stimulation)) OR TS=(Transcranial Electrical Stimulation)) OR TS=(Electrical Stimulation, Transcranial)) OR TS=(Electrical Stimulations, Transcranial)) OR TS=(Stimulation, Transcranial Electrical)) OR TS=(Stimulations, Transcranial Electrical)) OR TS=(Transcranial Electrical Stimulations)) OR TS=(Anodal Stimulation Transcranial Direct Current Stimulation)) OR TS=(Anodal Stimulation tDCS)) OR TS=(Anodal Stimulation tDCSs)) OR TS=(Stimulation tDCS, Anodal)) OR TS=(Stimulation tDCSs, Anodal)) OR TS=(tDCS, Anodal Stimulation)) OR TS=(tDCSs, Anodal Stimulation)) OR TS=(Repetitive Transcranial Electrical Stimulation) Date of operation: Sat Apr 1 2023 15:06:41 GMT+0800 (China Standard Time) Search Results: 14605

6: (((((((TS=(Virtual Reality Exposure Therapy)) OR TS=(Virtual Reality Immersion Therapy)) OR TS=(Virtual Reality Therapy)) OR TS=(Reality Therapies, Virtual)) OR TS=(Reality Therapy, Virtual)) OR TS=(Therapies, Virtual Reality)) OR TS=(Therapy, Virtual Reality)) OR TS=(Virtual Reality Therapies) Date of operation: Sat Apr 1 2023 15:08:55 GMT+0800 (China Standard Time) Search Results: 9316

7: ((((TS=( family intervention)) OR TS=(counseling)) OR TS=(Mindfulness)) OR TS=(acupuncture)) OR TS=(nursing) Date of operation: Sat Apr 1 2023 15:09:33 GMT+0800 (China Standard Time) Search Results: 1381276

8: (((((((((TS=(sandplay therapy )) OR TS=(Play Therapies)) OR TS=(Therapies, Play)) OR TS=(Therapy, Play)) OR TS=(Sandplay Therapy)) OR TS=(Sandplay Therapies)) OR TS=(Therapies, Sandplay)) OR TS=(Therapy, Sandplay)) OR TS=(Sandplay)) OR TS=(Sandplays) Date of operation: Sat Apr 1 2023 15:10:41 GMT+0800 (China Standard Time) Search Results: 370025

9: (TS=(Psychology, Positive)) OR TS=(Positive Psychology) Date of operation: Sat Apr 1 2023 15:11:11 GMT+0800 (China Standard Time) Search Results: 183186

10: #9 OR #8 OR #7 OR #6 OR #5 OR #4 OR #3 Date of operation: Sat Apr 1 2023 15:17:03 GMT+0800 (China Standard Time) Search Results: 3715008

11: #1 AND #2 AND #10 Date of operation: Sat Apr 1 2023 15:17:14 GMT+0800 (China Standard Time) Search Results: 24137

**Cochrane library**

#1 MeSH descriptor: [Internet Addiction Disorder] explode all trees

#2 (Addiction Disorder, Internet):ti,ab,kw OR (Addiction Disorders, Internet):ti,ab,kw OR (Disorder, Internet Addiction):ti,ab,kw OR (Disorders, Internet Addiction):ti,ab,kw OR (Internet Addiction Disorders):ti,ab,kw (Word variations have been searched)

#3 (Internet Addiction):ti,ab,kw OR (Addiction, Internet):ti,ab,kw OR (Addictions, Internet):ti,ab,kw OR (Internet Addictions):ti,ab,kw OR (Social Media Addiction):ti,ab,kw (Word variations have been searched)

#4 (Addiction, Social Media):ti,ab,kw OR (Addictions, Social Media):ti,ab,kw OR (Media Addiction, Social):ti,ab,kw OR (Media Addictions, Social):ti,ab,kw OR (Social Media Addictions):ti,ab,kw (Word variations have been searched)

#5 (Smartphone Addiction):ti,ab,kw OR (Addiction, Smartphone):ti,ab,kw OR (Addictions, Smartphone):ti,ab,kw OR (Smartphone Addictions):ti,ab,kw OR (Internet Gaming Disorder):ti,ab,kw (Word variations have been searched)

#6 (Disorder, Internet Gaming):ti,ab,kw OR (Disorders, Internet Gaming):ti,ab,kw OR (Gaming Disorder, Internet):ti,ab,kw OR (Gaming Disorders, Internet):ti,ab,kw OR (Internet Gaming Disorders):ti,ab,kw (Word variations have been searched)

#7 (problematic Internet use):ti,ab,kw OR (internet use disorder):ti,ab,kw OR (internet use):ti,ab,kw OR (Internet Uses):ti,ab,kw OR (Use, Internet):ti,ab,kw (Word variations have been searched)

#8 (Web Usage):ti,ab,kw OR (Usage, Web):ti,ab,kw OR (Web Use):ti,ab,kw OR (Use, Web):ti,ab,kw OR (Internet Usage):ti,ab,kw (Word variations have been searched)

#9 (Usage, Internet):ti,ab,kw OR (Internet-addicted):ti,ab,kw OR (Internet depend):ti,ab,kw OR (Internet overuse):ti,ab,kw OR (Compulsive internet):ti,ab,kw (Word variations have been searched)

#10 (Pathological internet):ti,ab,kw OR (Excessive internet):ti,ab,kw OR (Addictive internet):ti,ab,kw OR (Video game addiction):ti,ab,kw OR (Online game addiction):ti,ab,kw (Word variations have been searched)

#11 (Cybersex addiction):ti,ab,kw OR (Internet sex addiction):ti,ab,kw OR (Social network addiction):ti,ab,kw OR (Facebook addiction):ti,ab,kw (Word variations have been searched)

#12 #1 or #2 or #3 or #4 or #5 or #6 or #7 or #8 or #9 or #10 or #11

#13 MeSH descriptor: [Cognitive Behavioral Therapy] explode all trees

#14 (Cognitive Behaviour Therapy):ti,ab,kw OR (Behaviour Therapies, Cognitive):ti,ab,kw OR (Behaviour Therapy, Cognitive):ti,ab,kw OR (Cognitive Behaviour Therapies):ti,ab,kw AND (Therapies, Cognitive Behaviour):ti,ab,kw (Word variations have been searched)

#15 (Therapy, Cognitive Behaviour):ti,ab,kw OR (Cognitive Psychotherapy):ti,ab,kw OR (Cognitive Psychotherapies):ti,ab,kw OR (Psychotherapies, Cognitive):ti,ab,kw AND (Cognition Therapy):ti,ab,kw (Word variations have been searched)

#16 (Cognition Therapies):ti,ab,kw OR (Therapies, Cognition):ti,ab,kw OR (Therapy, Cognitive Behavior):ti,ab,kw OR (Behavior Therapies, Cognitive):ti,ab,kw AND (Cognitive Behavior Therapies):ti,ab,kw (Word variations have been searched)

#17 (Behavior Therapies, Cognitive):ti,ab,kw OR (Cognitive Behavior Therapies):ti,ab,kw OR (Therapies, Cognitive Behavior):ti,ab,kw OR (Therapy, Cognition):ti,ab,kw OR (Behavior Therapy, Cognitive):ti,ab,kw (Word variations have been searched)

#18 #13 or #14 or #15 or #16 or #17

#19 (Virtual Reality Exposure Therapy):ti,ab,kw OR (Virtual Reality Immersion Therapy):ti,ab,kw OR (Virtual Reality Therapy):ti,ab,kw OR (Reality Therapies, Virtual):ti,ab,kw OR (Reality Therapy, Virtual):ti,ab,kw (Word variations have been searched)

#20 (Therapies, Virtual Reality):ti,ab,kw OR (Therapy, Virtual Reality):ti,ab,kw OR (Virtual Reality Therapies):ti,ab,kw OR (Reality Therapies, Virtual):ti,ab,kw OR (Reality Therapy, Virtual):ti,ab,kw (Word variations have been searched)

#21 #19 or #20

#22 MeSH descriptor: [Exercise] explode all trees

#23 (Exercise, Physical):ti,ab,kw OR (Exercises, Physical):ti,ab,kw OR (Physical Exercise):ti,ab,kw OR (Physical Exercises):ti,ab,kw OR (Acute Exercise):ti,ab,kw (Word variations have been searched)

#24 (Acute Exercises):ti,ab,kw OR (Exercise, Acute):ti,ab,kw OR (Exercises, Acute):ti,ab,kw OR (Exercise, Isometric):ti,ab,kw OR (Exercises, Isometric):ti,ab,kw (Word variations have been searched)

#25 (football):ti,ab,kw OR (Tai Chi):ti,ab,kw OR (basketball):ti,ab,kw OR (High-Intensity Interval Training):ti,ab,kw OR (High Intensity Interval Training):ti,ab,kw (Word variations have been searched)

#26 (High-Intensity Interval Trainings):ti,ab,kw OR (Interval Training, High-Intensity):ti,ab,kw OR (Interval Trainings, High-Intensity):ti,ab,kw OR (Training, High-Intensity Interval):ti,ab,kw OR (Trainings, High-Intensity Interval):ti,ab,kw (Word variations have been searched)

#27 (High-Intensity Intermittent Exercise):ti,ab,kw OR (Exercise, High-Intensity Intermittent):ti,ab,kw OR (Exercises, High-Intensity Intermittent):ti,ab,kw OR (High-Intensity Intermittent Exercises):ti,ab,kw OR (Sprint Interval Training):ti,ab,kw (Word variations have been searched)

#28 #22 or #23 or #24 or #25 or #26 or #27

#29 (family intervention):ti,ab,kw OR (counseling):ti,ab,kw OR (Mindfulness):ti,ab,kw OR (acupuncture):ti,ab,kw OR (nursing):ti,ab,kw (Word variations have been searched)

#30 MeSH descriptor: [Play Therapy] explode all trees

#31 (Therapies, Sandplay):ti,ab,kw OR (Therapy, Sandplay):ti,ab,kw OR (Sandplay):ti,ab,kw OR (Sandplays):ti,ab,kw (Word variations have been searched)

#32 MeSH descriptor: [Transcranial Direct Current Stimulation] explode all trees

#33 (tDCS):ti,ab,kw OR (Cathodal Stimulation Transcranial Direct Current Stimulation):ti,ab,kw OR (Cathodal Stimulation tDCS):ti,ab,kw OR (Cathodal Stimulation tDCSs):ti,ab,kw OR (Stimulation tDCS, Cathodal):ti,ab,kw (Word variations have been searched)

#34 (Stimulation tDCSs, Anodal):ti,ab,kw OR (tDCS, Anodal Stimulation):ti,ab,kw OR (tDCSs, Anodal Stimulation):ti,ab,kw OR (Repetitive Transcranial Electrical Stimulation):ti,ab,kw (Word variations have been searched)

#35 #30 or #31 or #32 or #33 or #34

#36 MeSH descriptor: [Psychology, Positive] explode all trees

#37 (Positive Psychology):ti,ab,kw (Word variations have been searched)

#38 #36 or #37

#39 #18 or #21 or #28 or #29 or #35 or #38

#40 #12 and #39

**PubMed** **Search Strategy**

#1 "Internet Addiction Disorder"[Mesh]

#2 ((((((((((((((((((((((((((((((((((((((((((((((((Addiction Disorder, Internet[Title/Abstract]) OR (Addiction Disorders, Internet[Title/Abstract])) OR (Disorder, Internet Addiction[Title/Abstract])) OR (Disorders, Internet Addiction[Title/Abstract])) OR (Internet Addiction Disorders[Title/Abstract])) OR (Internet Addiction[Title/Abstract])) OR (Addiction, Internet[Title/Abstract])) OR (Addictions, Internet[Title/Abstract])) OR (Internet Addictions[Title/Abstract])) OR (Social Media Addiction[Title/Abstract])) OR (Addiction, Social Media[Title/Abstract])) OR (Addictions, Social Media[Title/Abstract])) OR (Media Addiction, Social[Title/Abstract])) OR (Media Addictions, Social[Title/Abstract])) OR (Social Media Addictions[Title/Abstract])) OR (Smartphone Addiction[Title/Abstract])) OR (Addiction, Smartphone[Title/Abstract])) OR (Addictions, Smartphone[Title/Abstract])) OR (Smartphone Addictions[Title/Abstract])) OR (Internet Gaming Disorder[Title/Abstract])) OR (Disorder, Internet Gaming[Title/Abstract])) OR (Disorders, Internet Gaming[Title/Abstract])) OR (Gaming Disorder, Internet[Title/Abstract])) OR (Gaming Disorders, Internet[Title/Abstract])) OR (Internet Gaming Disorders[Title/Abstract])) OR (problematic Internet use[Title/Abstract])) OR (internet use disorder[Title/Abstract])) OR (internet use[Title/Abstract])) OR (Internet Uses[Title/Abstract])) OR (Use, Internet[Title/Abstract])) OR (Web Usage[Title/Abstract])) OR (Usage, Web[Title/Abstract])) OR (Web Use[Title/Abstract])) OR (Use, Web[Title/Abstract])) OR (Internet Usage[Title/Abstract])) OR (Usage, Internet[Title/Abstract])) OR (Internet-addicted[Title/Abstract])) OR (Internet depend[Title/Abstract])) OR (Internet overuse[Title/Abstract])) OR (Compulsive internet[Title/Abstract])) OR (Pathological internet[Title/Abstract])) OR (Excessive internet[Title/Abstract])) OR (Addictive internet[Title/Abstract])) OR (Video game addiction[Title/Abstract])) OR (Online game addiction[Title/Abstract])) OR (Cybersex addiction[Title/Abstract])) OR (Internet sex addiction[Title/Abstract])) OR (Social network addiction[Title/Abstract])) OR (Facebook addiction[Title/Abstract])

#3 ("Internet Addiction Disorder"[Mesh]) OR (((((((((((((((((((((((((((((((((((((((((((((((((Addiction Disorder, Internet[Title/Abstract]) OR (Addiction Disorders, Internet[Title/Abstract])) OR (Disorder, Internet Addiction[Title/Abstract])) OR (Disorders, Internet Addiction[Title/Abstract])) OR (Internet Addiction Disorders[Title/Abstract])) OR (Internet Addiction[Title/Abstract])) OR (Addiction, Internet[Title/Abstract])) OR (Addictions, Internet[Title/Abstract])) OR (Internet Addictions[Title/Abstract])) OR (Social Media Addiction[Title/Abstract])) OR (Addiction, Social Media[Title/Abstract])) OR (Addictions, Social Media[Title/Abstract])) OR (Media Addiction, Social[Title/Abstract])) OR (Media Addictions, Social[Title/Abstract])) OR (Social Media Addictions[Title/Abstract])) OR (Smartphone Addiction[Title/Abstract])) OR (Addiction, Smartphone[Title/Abstract])) OR (Addictions, Smartphone[Title/Abstract])) OR (Smartphone Addictions[Title/Abstract])) OR (Internet Gaming Disorder[Title/Abstract])) OR (Disorder, Internet Gaming[Title/Abstract])) OR (Disorders, Internet Gaming[Title/Abstract])) OR (Gaming Disorder, Internet[Title/Abstract])) OR (Gaming Disorders, Internet[Title/Abstract])) OR (Internet Gaming Disorders[Title/Abstract])) OR (problematic Internet use[Title/Abstract])) OR (internet use disorder[Title/Abstract])) OR (internet use[Title/Abstract])) OR (Internet Uses[Title/Abstract])) OR (Use, Internet[Title/Abstract])) OR (Web Usage[Title/Abstract])) OR (Usage, Web[Title/Abstract])) OR (Web Use[Title/Abstract])) OR (Use, Web[Title/Abstract])) OR (Internet Usage[Title/Abstract])) OR (Usage, Internet[Title/Abstract])) OR (Internet-addicted[Title/Abstract])) OR (Internet depend[Title/Abstract])) OR (Internet overuse[Title/Abstract])) OR (Compulsive internet[Title/Abstract])) OR (Pathological internet[Title/Abstract])) OR (Excessive internet[Title/Abstract])) OR (Addictive internet[Title/Abstract])) OR (Video game addiction[Title/Abstract])) OR (Online game addiction[Title/Abstract])) OR (Cybersex addiction[Title/Abstract])) OR (Internet sex addiction[Title/Abstract])) OR (Social network addiction[Title/Abstract])) OR (Facebook addiction[Title/Abstract]))

#4 (((((((((((((((((((((((((((((Cognitive Behavioral Therapy[Title/Abstract]) OR (Behavioral Therapies, Cognitive[Title/Abstract])) OR (Behavioral Therapy, Cognitive[Title/Abstract])) OR (Cognitive Behavioral Therapies[Title/Abstract])) OR (Therapies, Cognitive Behavioral[Title/Abstract])) OR (Therapy, Cognitive Behavioral[Title/Abstract])) OR (Psychotherapy, Cognitive[Title/Abstract])) OR (Therapy, Cognitive[Title/Abstract])) OR (Cognitive Therapies[Title/Abstract])) OR (Therapies, Cognitive[Title/Abstract])) OR (Cognitive Therapy[Title/Abstract])) OR (Cognitive Behaviour Therapy[Title/Abstract])) OR (Behaviour Therapies, Cognitive[Title/Abstract])) OR (Behaviour Therapy, Cognitive[Title/Abstract])) OR (Cognitive Behaviour Therapies[Title/Abstract])) OR (Therapies, Cognitive Behaviour[Title/Abstract])) OR (Therapy, Cognitive Behaviour[Title/Abstract])) OR (Cognitive Psychotherapy[Title/Abstract])) OR (Cognitive Psychotherapies[Title/Abstract])) OR (Psychotherapies, Cognitive[Title/Abstract])) OR (Cognition Therapy[Title/Abstract])) OR (Cognition Therapies[Title/Abstract])) OR (Therapies, Cognition[Title/Abstract])) OR (Therapy, Cognitive Behavior[Title/Abstract])) OR (Behavior Therapies, Cognitive[Title/Abstract])) OR (Cognitive Behavior Therapies[Title/Abstract])) OR (Therapies, Cognitive Behavior[Title/Abstract])) OR (Therapy, Cognition[Title/Abstract])) OR (Behavior Therapy, Cognitive[Title/Abstract])) OR (Cognitive Behavior Therapy[Title/Abstract])

#5 (((((((Virtual Reality Exposure Therapy[MeSH Terms]) OR (Virtual Reality Immersion Therapy[Title/Abstract])) OR (Virtual Reality Therapy[Title/Abstract])) OR (Reality Therapies, Virtual[Title/Abstract])) OR (Reality Therapy, Virtual[Title/Abstract])) OR (Therapies, Virtual Reality[Title/Abstract])) OR (Therapy, Virtual Reality[Title/Abstract])) OR (Virtual Reality Therapies[Title/Abstract])

#6 ((((family intervention[Title/Abstract]) OR (counseling[Title/Abstract])) OR (Mindfulness[Title/Abstract])) OR (acupuncture[Title/Abstract])) OR (nursing[Title/Abstract])

#7 (((((((((sandplay therapy[MeSH Terms]) OR (Play Therapies[Title/Abstract])) OR (Therapies, Play[Title/Abstract])) OR (Therapy, Play[Title/Abstract])) OR (Sandplay Therapy[Title/Abstract])) OR (Sandplay Therapies[Title/Abstract])) OR (Therapies, Sandplay[Title/Abstract])) OR (Therapy, Sandplay[Title/Abstract])) OR (Sandplay[Title/Abstract])) OR (Sandplays[Title/Abstract])

#8 (((((((((((((((((((((((((((((((((((((((((exercise[MeSH Terms]) OR (Exercises[Title/Abstract])) OR (Physical Activity[Title/Abstract])) OR (Activities, Physical[Title/Abstract])) OR (Activity, Physical[Title/Abstract])) OR (Physical Activities[Title/Abstract])) OR (Exercise, Physical[Title/Abstract])) OR (Exercises, Physical[Title/Abstract])) OR (Physical Exercise[Title/Abstract])) OR (Physical Exercises[Title/Abstract])) OR (Acute Exercise[Title/Abstract])) OR (Acute Exercises[Title/Abstract])) OR (Exercise, Acute[Title/Abstract])) OR (Exercises, Acute[Title/Abstract])) OR (Exercise, Isometric[Title/Abstract])) OR (Exercises, Isometric[Title/Abstract])) OR (Isometric Exercises[Title/Abstract])) OR (Isometric Exercise[Title/Abstract])) OR (Exercise, Aerobic[Title/Abstract])) OR (Aerobic Exercise[Title/Abstract])) OR (Aerobic Exercises[Title/Abstract])) OR (Exercises, Aerobic[Title/Abstract])) OR (Exercise Training[Title/Abstract])) OR (Exercise Trainings[Title/Abstract])) OR (Training, Exercise[Title/Abstract])) OR (Trainings, Exercise[Title/Abstract])) OR (football[Title/Abstract])) OR (Tai Chi[Title/Abstract])) OR (basketball[Title/Abstract])) OR (High-Intensity Interval Training[Title/Abstract])) OR (High Intensity Interval Training[Title/Abstract])) OR (High-Intensity Interval Trainings[Title/Abstract])) OR (Interval Training, High-Intensity[Title/Abstract])) OR (Interval Trainings, High-Intensity[Title/Abstract])) OR (Training, High-Intensity Interval[Title/Abstract])) OR (Trainings, High-Intensity Interval[Title/Abstract])) OR (High-Intensity Intermittent Exercise[Title/Abstract])) OR (Exercise, High-Intensity Intermittent[Title/Abstract])) OR (Exercises, High-Intensity Intermittent[Title/Abstract])) OR (High-Intensity Intermittent Exercises[Title/Abstract])) OR (Sprint Interval Training[Title/Abstract])) OR (Sprint Interval Trainings[Title/Abstract])

#9 (((((((((((((((((((((((tDCS[MeSH Terms]) OR (Cathodal Stimulation Transcranial Direct Current Stimulation[Title/Abstract])) OR (Cathodal Stimulation tDCS[Title/Abstract])) OR (Cathodal Stimulation tDCSs[Title/Abstract])) OR (Stimulation tDCS, Cathodal[Title/Abstract])) OR (Stimulation tDCSs, Cathodal[Title/Abstract])) OR (tDCS, Cathodal Stimulation[Title/Abstract])) OR (tDCSs, Cathodal Stimulation[Title/Abstract])) OR (Transcranial Random Noise Stimulation[Title/Abstract])) OR (Transcranial Alternating Current Stimulation[Title/Abstract])) OR (Transcranial Electrical Stimulation[Title/Abstract])) OR (Electrical Stimulation, Transcranial[Title/Abstract])) OR (Electrical Stimulations, Transcranial[Title/Abstract])) OR (Stimulation, Transcranial Electrical[Title/Abstract])) OR (Stimulations, Transcranial Electrical[Title/Abstract])) OR (Transcranial Electrical Stimulations[Title/Abstract])) OR (Anodal Stimulation Transcranial Direct Current Stimulation[Title/Abstract])) OR (Anodal Stimulation tDCS[Title/Abstract])) OR (Anodal Stimulation tDCSs[Title/Abstract])) OR (Stimulation tDCS, Anodal[Title/Abstract])) OR (Stimulation tDCSs, Anodal[Title/Abstract])) OR (tDCS, Anodal Stimulation[Title/Abstract])) OR (tDCSs, Anodal Stimulation[Title/Abstract])) OR (Repetitive Transcranial Electrical Stimulation[Title/Abstract])

#10 (Psychology, Positive[MeSH Terms]) OR (Positive Psychology[Title/Abstract])

#11 (randomized controlled trial[pt] OR controlled clinical trial[pt] OR randomized[tiab] OR placebo[tiab] OR clinical trials as topic[mesh:noexp] OR randomly[tiab] OR trial[ti]) NOT (animals [mh] NOT (humans [mh] AND animals[mh]))

#12 (((((((Psychology, Positive[MeSH Terms]) OR (Positive Psychology[Title/Abstract])) OR ((((((((((((((((((((((((tDCS[MeSH Terms]) OR (Cathodal Stimulation Transcranial Direct Current Stimulation[Title/Abstract])) OR (Cathodal Stimulation tDCS[Title/Abstract])) OR (Cathodal Stimulation tDCSs[Title/Abstract])) OR (Stimulation tDCS, Cathodal[Title/Abstract])) OR (Stimulation tDCSs, Cathodal[Title/Abstract])) OR (tDCS, Cathodal Stimulation[Title/Abstract])) OR (tDCSs, Cathodal Stimulation[Title/Abstract])) OR (Transcranial Random Noise Stimulation[Title/Abstract])) OR (Transcranial Alternating Current Stimulation[Title/Abstract])) OR (Transcranial Electrical Stimulation[Title/Abstract])) OR (Electrical Stimulation, Transcranial[Title/Abstract])) OR (Electrical Stimulations, Transcranial[Title/Abstract])) OR (Stimulation, Transcranial Electrical[Title/Abstract])) OR (Stimulations, Transcranial Electrical[Title/Abstract])) OR (Transcranial Electrical Stimulations[Title/Abstract])) OR (Anodal Stimulation Transcranial Direct Current Stimulation[Title/Abstract])) OR (Anodal Stimulation tDCS[Title/Abstract])) OR (Anodal Stimulation tDCSs[Title/Abstract])) OR (Stimulation tDCS, Anodal[Title/Abstract])) OR (Stimulation tDCSs, Anodal[Title/Abstract])) OR (tDCS, Anodal Stimulation[Title/Abstract])) OR (tDCSs, Anodal Stimulation[Title/Abstract])) OR (Repetitive Transcranial Electrical Stimulation[Title/Abstract]))) OR ((((((((((((((((((((((((((((((((((((((((((exercise[MeSH Terms]) OR (Exercises[Title/Abstract])) OR (Physical Activity[Title/Abstract])) OR (Activities, Physical[Title/Abstract])) OR (Activity, Physical[Title/Abstract])) OR (Physical Activities[Title/Abstract])) OR (Exercise, Physical[Title/Abstract])) OR (Exercises, Physical[Title/Abstract])) OR (Physical Exercise[Title/Abstract])) OR (Physical Exercises[Title/Abstract])) OR (Acute Exercise[Title/Abstract])) OR (Acute Exercises[Title/Abstract])) OR (Exercise, Acute[Title/Abstract])) OR (Exercises, Acute[Title/Abstract])) OR (Exercise, Isometric[Title/Abstract])) OR (Exercises, Isometric[Title/Abstract])) OR (Isometric Exercises[Title/Abstract])) OR (Isometric Exercise[Title/Abstract])) OR (Exercise, Aerobic[Title/Abstract])) OR (Aerobic Exercise[Title/Abstract])) OR (Aerobic Exercises[Title/Abstract])) OR (Exercises, Aerobic[Title/Abstract])) OR (Exercise Training[Title/Abstract])) OR (Exercise Trainings[Title/Abstract])) OR (Training, Exercise[Title/Abstract])) OR (Trainings, Exercise[Title/Abstract])) OR (football[Title/Abstract])) OR (Tai Chi[Title/Abstract])) OR (basketball[Title/Abstract])) OR (High-Intensity Interval Training[Title/Abstract])) OR (High Intensity Interval Training[Title/Abstract])) OR (High-Intensity Interval Trainings[Title/Abstract])) OR (Interval Training, High-Intensity[Title/Abstract])) OR (Interval Trainings, High-Intensity[Title/Abstract])) OR (Training, High-Intensity Interval[Title/Abstract])) OR (Trainings, High-Intensity Interval[Title/Abstract])) OR (High-Intensity Intermittent Exercise[Title/Abstract])) OR (Exercise, High-Intensity Intermittent[Title/Abstract])) OR (Exercises, High-Intensity Intermittent[Title/Abstract])) OR (High-Intensity Intermittent Exercises[Title/Abstract])) OR (Sprint Interval Training[Title/Abstract])) OR (Sprint Interval Trainings[Title/Abstract]))) OR ((((((((((sandplay therapy[MeSH Terms]) OR (Play Therapies[Title/Abstract])) OR (Therapies, Play[Title/Abstract])) OR (Therapy, Play[Title/Abstract])) OR (Sandplay Therapy[Title/Abstract])) OR (Sandplay Therapies[Title/Abstract])) OR (Therapies, Sandplay[Title/Abstract])) OR (Therapy, Sandplay[Title/Abstract])) OR (Sandplay[Title/Abstract])) OR (Sandplays[Title/Abstract]))) OR (((((family intervention[Title/Abstract]) OR (counseling[Title/Abstract])) OR (Mindfulness[Title/Abstract])) OR (acupuncture[Title/Abstract])) OR (nursing[Title/Abstract]))) OR ((((((((Virtual Reality Exposure Therapy[MeSH Terms]) OR (Virtual Reality Immersion Therapy[Title/Abstract])) OR (Virtual Reality Therapy[Title/Abstract])) OR (Reality Therapies, Virtual[Title/Abstract])) OR (Reality Therapy, Virtual[Title/Abstract])) OR (Therapies, Virtual Reality[Title/Abstract])) OR (Therapy, Virtual Reality[Title/Abstract])) OR (Virtual Reality Therapies[Title/Abstract]))) OR ((((((((((((((((((((((((((((((Cognitive Behavioral Therapy[Title/Abstract]) OR (Behavioral Therapies, Cognitive[Title/Abstract])) OR (Behavioral Therapy, Cognitive[Title/Abstract])) OR (Cognitive Behavioral Therapies[Title/Abstract])) OR (Therapies, Cognitive Behavioral[Title/Abstract])) OR (Therapy, Cognitive Behavioral[Title/Abstract])) OR (Psychotherapy, Cognitive[Title/Abstract])) OR (Therapy, Cognitive[Title/Abstract])) OR (Cognitive Therapies[Title/Abstract])) OR (Therapies, Cognitive[Title/Abstract])) OR (Cognitive Therapy[Title/Abstract])) OR (Cognitive Behaviour Therapy[Title/Abstract])) OR (Behaviour Therapies, Cognitive[Title/Abstract])) OR (Behaviour Therapy, Cognitive[Title/Abstract])) OR (Cognitive Behaviour Therapies[Title/Abstract])) OR (Therapies, Cognitive Behaviour[Title/Abstract])) OR (Therapy, Cognitive Behaviour[Title/Abstract])) OR (Cognitive Psychotherapy[Title/Abstract])) OR (Cognitive Psychotherapies[Title/Abstract])) OR (Psychotherapies, Cognitive[Title/Abstract])) OR (Cognition Therapy[Title/Abstract])) OR (Cognition Therapies[Title/Abstract])) OR (Therapies, Cognition[Title/Abstract])) OR (Therapy, Cognitive Behavior[Title/Abstract])) OR (Behavior Therapies, Cognitive[Title/Abstract])) OR (Cognitive Behavior Therapies[Title/Abstract])) OR (Therapies, Cognitive Behavior[Title/Abstract])) OR (Therapy, Cognition[Title/Abstract])) OR (Behavior Therapy, Cognitive[Title/Abstract])) OR (Cognitive Behavior Therapy[Title/Abstract]))

#13 (randomized controlled trial [pt] OR controlled clinical trial [pt] OR randomized [tiab] OR placebo [tiab] OR drug therapy [sh] OR randomly [tiab] OR trial [tiab] OR groups [tiab]) NOT (animals [mh] NOT humans [mh])

#14 (((randomized controlled trial [pt] OR controlled clinical trial [pt] OR randomized [tiab] OR placebo [tiab] OR drug therapy [sh] OR randomly [tiab] OR trial [tiab] OR groups [tiab]) NOT (animals [mh] NOT humans [mh])) AND ((((((((Psychology, Positive[MeSH Terms]) OR (Positive Psychology[Title/Abstract])) OR ((((((((((((((((((((((((tDCS[MeSH Terms]) OR (Cathodal Stimulation Transcranial Direct Current Stimulation[Title/Abstract])) OR (Cathodal Stimulation tDCS[Title/Abstract])) OR (Cathodal Stimulation tDCSs[Title/Abstract])) OR (Stimulation tDCS, Cathodal[Title/Abstract])) OR (Stimulation tDCSs, Cathodal[Title/Abstract])) OR (tDCS, Cathodal Stimulation[Title/Abstract])) OR (tDCSs, Cathodal Stimulation[Title/Abstract])) OR (Transcranial Random Noise Stimulation[Title/Abstract])) OR (Transcranial Alternating Current Stimulation[Title/Abstract])) OR (Transcranial Electrical Stimulation[Title/Abstract])) OR (Electrical Stimulation, Transcranial[Title/Abstract])) OR (Electrical Stimulations, Transcranial[Title/Abstract])) OR (Stimulation, Transcranial Electrical[Title/Abstract])) OR (Stimulations, Transcranial Electrical[Title/Abstract])) OR (Transcranial Electrical Stimulations[Title/Abstract])) OR (Anodal Stimulation Transcranial Direct Current Stimulation[Title/Abstract])) OR (Anodal Stimulation tDCS[Title/Abstract])) OR (Anodal Stimulation tDCSs[Title/Abstract])) OR (Stimulation tDCS, Anodal[Title/Abstract])) OR (Stimulation tDCSs, Anodal[Title/Abstract])) OR (tDCS, Anodal Stimulation[Title/Abstract])) OR (tDCSs, Anodal Stimulation[Title/Abstract])) OR (Repetitive Transcranial Electrical Stimulation[Title/Abstract]))) OR ((((((((((((((((((((((((((((((((((((((((((exercise[MeSH Terms]) OR (Exercises[Title/Abstract])) OR (Physical Activity[Title/Abstract])) OR (Activities, Physical[Title/Abstract])) OR (Activity, Physical[Title/Abstract])) OR (Physical Activities[Title/Abstract])) OR (Exercise, Physical[Title/Abstract])) OR (Exercises, Physical[Title/Abstract])) OR (Physical Exercise[Title/Abstract])) OR (Physical Exercises[Title/Abstract])) OR (Acute Exercise[Title/Abstract])) OR (Acute Exercises[Title/Abstract])) OR (Exercise, Acute[Title/Abstract])) OR (Exercises, Acute[Title/Abstract])) OR (Exercise, Isometric[Title/Abstract])) OR (Exercises, Isometric[Title/Abstract])) OR (Isometric Exercises[Title/Abstract])) OR (Isometric Exercise[Title/Abstract])) OR (Exercise, Aerobic[Title/Abstract])) OR (Aerobic Exercise[Title/Abstract])) OR (Aerobic Exercises[Title/Abstract])) OR (Exercises, Aerobic[Title/Abstract])) OR (Exercise Training[Title/Abstract])) OR (Exercise Trainings[Title/Abstract])) OR (Training, Exercise[Title/Abstract])) OR (Trainings, Exercise[Title/Abstract])) OR (football[Title/Abstract])) OR (Tai Chi[Title/Abstract])) OR (basketball[Title/Abstract])) OR (High-Intensity Interval Training[Title/Abstract])) OR (High Intensity Interval Training[Title/Abstract])) OR (High-Intensity Interval Trainings[Title/Abstract])) OR (Interval Training, High-Intensity[Title/Abstract])) OR (Interval Trainings, High-Intensity[Title/Abstract])) OR (Training, High-Intensity Interval[Title/Abstract])) OR (Trainings, High-Intensity Interval[Title/Abstract])) OR (High-Intensity Intermittent Exercise[Title/Abstract])) OR (Exercise, High-Intensity Intermittent[Title/Abstract])) OR (Exercises, High-Intensity Intermittent[Title/Abstract])) OR (High-Intensity Intermittent Exercises[Title/Abstract])) OR (Sprint Interval Training[Title/Abstract])) OR (Sprint Interval Trainings[Title/Abstract]))) OR ((((((((((sandplay therapy[MeSH Terms]) OR (Play Therapies[Title/Abstract])) OR (Therapies, Play[Title/Abstract])) OR (Therapy, Play[Title/Abstract])) OR (Sandplay Therapy[Title/Abstract])) OR (Sandplay Therapies[Title/Abstract])) OR (Therapies, Sandplay[Title/Abstract])) OR (Therapy, Sandplay[Title/Abstract])) OR (Sandplay[Title/Abstract])) OR (Sandplays[Title/Abstract]))) OR (((((family intervention[Title/Abstract]) OR (counseling[Title/Abstract])) OR (Mindfulness[Title/Abstract])) OR (acupuncture[Title/Abstract])) OR (nursing[Title/Abstract]))) OR ((((((((Virtual Reality Exposure Therapy[MeSH Terms]) OR (Virtual Reality Immersion Therapy[Title/Abstract])) OR (Virtual Reality Therapy[Title/Abstract])) OR (Reality Therapies, Virtual[Title/Abstract])) OR (Reality Therapy, Virtual[Title/Abstract])) OR (Therapies, Virtual Reality[Title/Abstract])) OR (Therapy, Virtual Reality[Title/Abstract])) OR (Virtual Reality Therapies[Title/Abstract]))) OR ((((((((((((((((((((((((((((((Cognitive Behavioral Therapy[Title/Abstract]) OR (Behavioral Therapies, Cognitive[Title/Abstract])) OR (Behavioral Therapy, Cognitive[Title/Abstract])) OR (Cognitive Behavioral Therapies[Title/Abstract])) OR (Therapies, Cognitive Behavioral[Title/Abstract])) OR (Therapy, Cognitive Behavioral[Title/Abstract])) OR (Psychotherapy, Cognitive[Title/Abstract])) OR (Therapy, Cognitive[Title/Abstract])) OR (Cognitive Therapies[Title/Abstract])) OR (Therapies, Cognitive[Title/Abstract])) OR (Cognitive Therapy[Title/Abstract])) OR (Cognitive Behaviour Therapy[Title/Abstract])) OR (Behaviour Therapies, Cognitive[Title/Abstract])) OR (Behaviour Therapy, Cognitive[Title/Abstract])) OR (Cognitive Behaviour Therapies[Title/Abstract])) OR (Therapies, Cognitive Behaviour[Title/Abstract])) OR (Therapy, Cognitive Behaviour[Title/Abstract])) OR (Cognitive Psychotherapy[Title/Abstract])) OR (Cognitive Psychotherapies[Title/Abstract])) OR (Psychotherapies, Cognitive[Title/Abstract])) OR (Cognition Therapy[Title/Abstract])) OR (Cognition Therapies[Title/Abstract])) OR (Therapies, Cognition[Title/Abstract])) OR (Therapy, Cognitive Behavior[Title/Abstract])) OR (Behavior Therapies, Cognitive[Title/Abstract])) OR (Cognitive Behavior Therapies[Title/Abstract])) OR (Therapies, Cognitive Behavior[Title/Abstract])) OR (Therapy, Cognition[Title/Abstract])) OR (Behavior Therapy, Cognitive[Title/Abstract])) OR (Cognitive Behavior Therapy[Title/Abstract])))) AND (("Internet Addiction Disorder"[Mesh]) OR (((((((((((((((((((((((((((((((((((((((((((((((((Addiction Disorder, Internet[Title/Abstract]) OR (Addiction Disorders, Internet[Title/Abstract])) OR (Disorder, Internet Addiction[Title/Abstract])) OR (Disorders, Internet Addiction[Title/Abstract])) OR (Internet Addiction Disorders[Title/Abstract])) OR (Internet Addiction[Title/Abstract])) OR (Addiction, Internet[Title/Abstract])) OR (Addictions, Internet[Title/Abstract])) OR (Internet Addictions[Title/Abstract])) OR (Social Media Addiction[Title/Abstract])) OR (Addiction, Social Media[Title/Abstract])) OR (Addictions, Social Media[Title/Abstract])) OR (Media Addiction, Social[Title/Abstract])) OR (Media Addictions, Social[Title/Abstract])) OR (Social Media Addictions[Title/Abstract])) OR (Smartphone Addiction[Title/Abstract])) OR (Addiction, Smartphone[Title/Abstract])) OR (Addictions, Smartphone[Title/Abstract])) OR (Smartphone Addictions[Title/Abstract])) OR (Internet Gaming Disorder[Title/Abstract])) OR (Disorder, Internet Gaming[Title/Abstract])) OR (Disorders, Internet Gaming[Title/Abstract])) OR (Gaming Disorder, Internet[Title/Abstract])) OR (Gaming Disorders, Internet[Title/Abstract])) OR (Internet Gaming Disorders[Title/Abstract])) OR (problematic Internet use[Title/Abstract])) OR (internet use disorder[Title/Abstract])) OR (internet use[Title/Abstract])) OR (Internet Uses[Title/Abstract])) OR (Use, Internet[Title/Abstract])) OR (Web Usage[Title/Abstract])) OR (Usage, Web[Title/Abstract])) OR (Web Use[Title/Abstract])) OR (Use, Web[Title/Abstract])) OR (Internet Usage[Title/Abstract])) OR (Usage, Internet[Title/Abstract])) OR (Internet-addicted[Title/Abstract])) OR (Internet depend[Title/Abstract])) OR (Internet overuse[Title/Abstract])) OR (Compulsive internet[Title/Abstract])) OR (Pathological internet[Title/Abstract])) OR (Excessive internet[Title/Abstract])) OR (Addictive internet[Title/Abstract])) OR (Video game addiction[Title/Abstract])) OR (Online game addiction[Title/Abstract])) OR (Cybersex addiction[Title/Abstract])) OR (Internet sex addiction[Title/Abstract])) OR (Social network addiction[Title/Abstract])) OR (Facebook addiction[Title/Abstract])))

**Embase Search Strategy**

#11. #1 AND #2 AND #10 1,370

1 Apr 2023

#10. #3 OR #4 OR #5 OR #6 OR #7 OR #8 OR #9 4,092,985 1 Apr 2023

#9. 'psychology, positive'/exp OR 'psychology, 115,657 1 Apr 2023

positive' OR (('psychology,'/exp OR psychology,)

AND positive) OR 'positive psychology':ti,ab,kw

#8. 'sandplay therapy' OR (sandplay AND 422 1 Apr 2023

('therapy'/exp OR therapy)) OR 'play

therapies':ti,ab,kw OR 'therapies, play':ti,ab,kw

OR 'therapy, play':ti,ab,kw OR 'sandplay

therapy':ti,ab,kw OR 'sandplay

therapies':ti,ab,kw OR 'therapies,

sandplay':ti,ab,kw OR 'therapy,

sandplay':ti,ab,kw OR sandplay:ti,ab,kw OR

sandplays:ti,ab,kw

#7. 'family intervention'/exp OR 'family 611,977 1 Apr 2023

intervention' OR (('family'/exp OR family) AND

('intervention'/exp OR intervention)) OR

counseling:ti,ab,kw OR mindfulness:ti,ab,kw OR

acupuncture:ti,ab,kw OR nursing:ti,ab,kw

#6. 'virtual reality exposure therapy'/exp OR 3,159,503 1 Apr 2023

'virtual reality exposure therapy' OR (virtual

AND ('reality'/exp OR reality) AND

('exposure'/exp OR exposure) AND ('therapy'/exp

OR therapy)) OR 'virtual reality

immersion':ti,ab,kw OR therapy:ti,ab,kw OR

'virtual reality therapy':ti,ab,kw OR 'reality

therapies, virtual':ti,ab,kw OR 'reality therapy,

virtual':ti,ab,kw OR 'therapies, virtual

reality':ti,ab,kw OR 'therapy, virtual

reality':ti,ab,kw OR 'virtual reality

therapies':ti,ab,kw

#5. tdcs OR 'cathodal stimulation transcranial direct 10,257 1 Apr 2023

current stimulation':ti,ab,kw OR 'cathodal

stimulation tdcs':ti,ab,kw OR 'cathodal

stimulation tdcss':ti,ab,kw OR 'stimulation tdcs,

cathodal':ti,ab,kw OR 'stimulation tdcss,

cathodal':ti,ab,kw OR 'tdcs, cathodal

stimulation':ti,ab,kw OR 'tdcss, cathodal

stimulation':ti,ab,kw OR 'transcranial random

noise stimulation':ti,ab,kw OR 'transcranial

alternating current stimulation':ti,ab,kw OR

'transcranial electrical stimulation':ti,ab,kw OR

'electrical stimulation, transcranial':ti,ab,kw

OR 'electrical stimulations,

transcranial':ti,ab,kw OR 'stimulation,

transcranial electrical':ti,ab,kw OR

'stimulations, transcranial electrical':ti,ab,kw

OR 'transcranial electrical

stimulations':ti,ab,kw OR 'anodal stimulation

transcranial direct current stimulation':ti,ab,kw

OR 'anodal stimulation tdcs':ti,ab,kw OR 'anodal

stimulation tdcss':ti,ab,kw OR 'stimulation tdcs,

anodal':ti,ab,kw OR 'stimulation tdcss,

anodal':ti,ab,kw OR 'tdcs, anodal

stimulation':ti,ab,kw OR 'tdcss, anodal

stimulation':ti,ab,kw OR 'repetitive transcranial

electrical stimulation':ti,ab,kw

#4. exercises OR 'physical activity':ti,ab,kw OR 325,096 1 Apr 2023

'activities, physical':ti,ab,kw OR 'activity,

physical':ti,ab,kw OR 'physical

activities':ti,ab,kw OR 'exercise,

physical':ti,ab,kw OR 'exercises,

physical':ti,ab,kw OR 'physical

exercise':ti,ab,kw OR 'physical

exercises':ti,ab,kw OR 'acute exercise':ti,ab,kw

OR 'acute exercises':ti,ab,kw OR 'exercise,

acute':ti,ab,kw OR 'exercises, acute':ti,ab,kw OR

'exercise, isometric':ti,ab,kw OR 'exercises,

isometric':ti,ab,kw OR 'isometric

exercises':ti,ab,kw OR 'isometric

exercise':ti,ab,kw OR 'exercise,

aerobic':ti,ab,kw OR 'aerobic exercise':ti,ab,kw

OR 'aerobic exercises':ti,ab,kw OR 'exercises,

aerobic':ti,ab,kw OR 'exercise training':ti,ab,kw

OR 'exercise trainings':ti,ab,kw OR 'training,

exercise':ti,ab,kw OR 'trainings,

exercise':ti,ab,kw OR football:ti,ab,kw OR 'tai

chi':ti,ab,kw OR basketball:ti,ab,kw OR

'high-intensity interval training':ti,ab,kw OR

'high intensity interval training':ti,ab,kw OR

'high-intensity interval trainings':ti,ab,kw OR

'interval training, high-intensity':ti,ab,kw OR

'interval trainings, high-intensity':ti,ab,kw OR

'training, high-intensity interval':ti,ab,kw OR

'trainings, high-intensity interval':ti,ab,kw OR

'high-intensity intermittent exercise':ti,ab,kw

OR 'exercise, high-intensity

intermittent':ti,ab,kw OR 'exercises,

high-intensity intermittent':ti,ab,kw OR

'high-intensity intermittent exercises':ti,ab,kw

OR 'sprint interval training':ti,ab,kw OR 'sprint

interval trainings':ti,ab,kw

#3. 'behavioral therapies, cognitive' OR (behavioral 19,132 1 Apr 2023

AND therapies, AND cognitive) OR 'behavioral

therapy, cognitive':ti,ab,kw OR 'cognitive

behavioral therapies':ti,ab,kw OR 'therapies,

cognitive behavioral':ti,ab,kw OR 'therapy,

cognitive behavioral':ti,ab,kw OR 'psychotherapy,

cognitive':ti,ab,kw OR 'therapy,

cognitive':ti,ab,kw OR 'cognitive

therapies':ti,ab,kw OR 'therapies,

cognitive':ti,ab,kw OR 'cognitive

therapy':ti,ab,kw OR 'cognitive behaviour

therapy':ti,ab,kw OR 'behaviour therapies,

cognitive':ti,ab,kw OR 'behaviour therapy,

cognitive':ti,ab,kw OR 'cognitive behaviour

therapies':ti,ab,kw OR 'therapies, cognitive

behaviour':ti,ab,kw OR 'therapy, cognitive

behaviour':ti,ab,kw OR 'cognitive

psychotherapy':ti,ab,kw OR 'cognitive

psychotherapies':ti,ab,kw OR 'psychotherapies,

cognitive':ti,ab,kw OR 'cognition

therapy':ti,ab,kw OR 'cognition

therapies':ti,ab,kw OR 'therapies,

cognition':ti,ab,kw OR 'therapy, cognitive

behavior':ti,ab,kw OR 'behavior therapies,

cognitive':ti,ab,kw OR 'cognitive behavior

therapies':ti,ab,kw OR 'therapies, cognitive

behavior':ti,ab,kw OR 'therapy,

cognition':ti,ab,kw OR 'behavior therapy,

cognitive':ti,ab,kw OR 'cognitive behavior

therapy':ti,ab,kw

#2. 'internet addiction disorder'/exp OR 'internet 13,491 1 Apr 2023

addiction disorder' OR (('internet'/exp OR

internet) AND ('addiction'/exp OR addiction) AND

('disorder'/exp OR disorder)) OR 'addiction

disorder, internet':ti,ab,kw OR 'addiction

disorders, internet':ti,ab,kw OR 'disorder,

internet addiction':ti,ab,kw OR 'disorders,

internet addiction':ti,ab,kw OR 'internet

addiction disorders':ti,ab,kw OR 'internet

addiction':ti,ab,kw OR 'addiction,

internet':ti,ab,kw OR 'addictions,

internet':ti,ab,kw OR 'internet

addictions':ti,ab,kw OR 'social media

addiction':ti,ab,kw OR 'addiction, social

media':ti,ab,kw OR 'addictions, social

media':ti,ab,kw OR 'media addiction,

social':ti,ab,kw OR 'media addictions,

social':ti,ab,kw OR 'social media

addictions':ti,ab,kw OR 'smartphone

addiction':ti,ab,kw OR 'addiction,

smartphone':ti,ab,kw OR 'addictions,

smartphone':ti,ab,kw OR 'smartphone

addictions':ti,ab,kw OR 'internet gaming

disorder':ti,ab,kw OR 'disorder, internet

gaming':ti,ab,kw OR 'disorders, internet

gaming':ti,ab,kw OR 'gaming disorder,

internet':ti,ab,kw OR 'gaming disorders,

internet':ti,ab,kw OR 'internet gaming

disorders':ti,ab,kw OR 'problematic internet

use':ti,ab,kw OR 'internet use disorder':ti,ab,kw

OR 'internet use':ti,ab,kw OR 'internet

uses':ti,ab,kw OR 'use, internet':ti,ab,kw OR

'web usage':ti,ab,kw OR 'usage, web':ti,ab,kw OR

'web use':ti,ab,kw OR 'use, web':ti,ab,kw OR

'internet usage':ti,ab,kw OR 'usage,

internet':ti,ab,kw OR 'internet

addicted':ti,ab,kw OR 'internet depend':ti,ab,kw

OR 'internet overuse':ti,ab,kw OR 'compulsive

internet':ti,ab,kw OR 'pathological

internet':ti,ab,kw OR 'excessive

internet':ti,ab,kw OR 'addictive

internet':ti,ab,kw OR 'video game

addiction':ti,ab,kw OR 'online game

addiction':ti,ab,kw OR 'cybersex

addiction':ti,ab,kw OR 'internet sex

addiction':ti,ab,kw OR 'social network

addiction':ti,ab,kw OR 'facebook

addiction':ti,ab,kw

#1. 'randomized controlled trial'/exp OR 'randomized 6,331,028 1 Apr 2023

controlled trial' OR (randomized AND controlled

AND ('trial'/exp OR trial)) OR 'controlled

clinical trial':ti,ab,kw OR random:ti,ab,kw OR

placebo:ti,ab,kw OR 'clinical trials as

topic':ti,ab,kw OR trial:ti,ab,kw OR

intervention:ti,ab,kw OR treat:ti,ab,kw OR

therapy:ti,ab,kw OR program:ti,ab,kw OR

workshop:ti,ab,kw OR train:ti,ab,kw

**Chinese BioMedical Literature Search Strategy**

(cognitive behavioral therapy or behavioral therapy or exercise or exercise or physical activity or badminton or basketball or soccer exercise or Tai Chi or Baduanjin or high-intensity interval exercises or high-intensity interval training or aerobic exercise or resistance exercises or physical therapy or physical therapy or transcranial magnetic stimulation or electrical stimulation or family therapy or group intervention or family intervention or psychological therapy or counseling or positive thinking or acupuncture or nursing or positive psychotherapy or positive psychological intervention or sandplay or sandbox therapy or virtual reality therapy or reality therapy or hypnosis) and (Internet addiction or Internet addiction or gaming addiction or computer addiction or video game addiction or online gaming addiction or Internet addiction or transitional Internet access) and (random) 134 2023-04-1 23:04:32.0

**China National Knowledge Infrastructure Search Strategy**

Search formula A: Topic = cognitive behavioral therapy + behavioral therapy + sports + exercise + physical activity + badminton + basketball + soccer + sports exercise + tai chi + bada-jin + high intensity interval exercises + OR Topic = electrical stimulation + family therapy + group intervention + family intervention + psychotherapy + counseling + positive thinking + acupuncture + nursing + positive psychotherapy + positive psychological intervention + sandplay + sandplay therapy + virtual reality Therapy + Reality Therapy + Hypnosis + Sandplay + Sandplay OR Topic = Virtual Reality Therapy + Reality Therapy + Hypnosis + Virtual Reality Techniques AND Topic = Internet Addiction + Internet Addiction + Gaming Addiction + Computer Addiction + Video Game Addiction + Online Game Addiction + Internet Addiction + Transitional Internet Access OR ( Title = Cognitive Behavioral Therapy + Behavioral Therapy + Exercise + Exercise + Physical Activity + Badminton + Basketball + soccer + exercise + tai chi + bajuanjin + high intensity interval exercises + or title = electrical stimulation + family therapy + group intervention + family intervention + psychotherapy + counseling + positive thinking + acupuncture + nursing + positive psychotherapy + positive psychological intervention + sandplay + sandplay therapy + virtual reality therapy + reality therapy + hypnosis + sandplay + sandplay therapy or title = virtual reality therapy + reality therapy + Hypnosis + Virtual Reality Therapy or title = Internet Addiction + Internet Addiction + Game Addiction + Computer Addiction + Video Game Addiction + Online Game Addiction + Internet Addiction + Transitional Internet Access ) (fuzzy match)

**WanFang** **Search Strategy**

(cognitive behavioral therapy or behavioral therapy or exercise or exercise or physical activity or badminton or basketball or soccer exercise or Tai Chi or Baduanjin or high-intensity interval exercises or high-intensity interval training or aerobic exercise or resistance exercises or physical therapy or physical therapy or transcranial magnetic stimulation or electrical stimulation or family therapy or group intervention or family intervention or psychological therapy or counseling or positive thinking or acupuncture or nursing or positive psychotherapy or positive psychological intervention or sandplay or sandbox therapy or virtual reality therapy or reality therapy or hypnosis) and (Internet addiction or Internet addiction or gaming addiction or computer addiction or video game addiction or online gaming addiction or Internet addiction or transitional Internet access) and (random)

**China Science and Technology Journal Search Strategy**

#1= Cognitive-behavioral therapy or behavioral therapy or exercise or exercise or physical activity or badminton or basketball or soccer exercise or Tai Chi or Baduanjin or high-intensity interval exercises or high-intensity interval training or aerobic exercise or resistance exercises or physical therapy or physical therapy or physical therapy or transcranial magnetic stimulation or electrical stimulation or family therapy or group intervention or family intervention or Psychotherapy or counseling or positive thinking or acupuncture or nursing or positive psychotherapy or positive psychological intervention or sandplay or sandbox therapy or virtual reality therapy or reality therapy or hypnosis

#2= Internet addiction or Internet addiction or gaming addiction or computer addiction or video game addiction or online game addiction or Internet addiction or transitioning to the Internet

#3= #1 and #2
