# Supplementary material for: Effects of non-pharmacological interventions on youth with internet addiction: a systematic review and meta-analysis of randomized controlled trials
Source: Front Psychiatry. 2024 Jan 11;14:1327200. doi: 10.3389/fpsyt.2023.1327200 (PMC10808612; doi:10.3389/fpsyt.2023.1327200)
Supplement: Supplementary file 7 [file Data_Sheet_3.docx]

**Qualified literature**

[1] AGBARIA Q. Cognitive behavioral intervention in dealing with Internet addiction among Arab teenagers in Israel [J]. International journal of mental health and addiction.

[2] BAI Y, FAN F. The Effects of Group Counseling on Internet Dependent College Students [J]. Chinese Mental Health Journal, 2007, (04): 247-50.

[3] CAO F, SU L, GAO X, et al. Control Study of Group Psychotherapy on Middle School Students with Internet Overuse [J]. Chinese Mental Health Journal, 2007, (05): 346-9+58.

[4] ZHENG W, YE M, LIN F, et al. Effect Analysis of Group Psychotherapy in College Students with Internet Addiction [J]. Medicine and Society, 2007, 20(6): 28-30.

[5] KIM J-U. The effect of a R/T group counseling program on the Internet addiction level and self-esteem of Internet addiction university students [J]. International Journal of reality therapy, 2008, 27(2).

[6] GUO M, YU F, CHAO X, et al. Impact Evaluation ofGroupCounseling onInternetAddictionAdolescents [J]. Chinese Journal of School Health, 2008, 29(1): 17-9.

[7] LI G, DAI X. control study of cognitive-bahavior or therapy in adolescents with internet addiction disorder [J]. Chinese Mental Health Journal, 2009, 23(7).

[8] LIAO X. Intervention of Aerobic Exercise for Internet Addiction College Students [J]. Journal of Hunan University of Technology, 2009, 23(6).

[9] ZHANG L. Intervention of group psychotherapy and physical exercise prescription for Internet addiction among college students [J]. Psychological science, 2009, 32(03): 738-41.

[10] ZHANG W, LU J. Research on group counseling of injecting emotional factors into Internet addicted College Students [J]. Psychological science, 2009, 32(03): 525-7.

[11] DU Y-S, JIANG W, VANCE A. Longer term effect of randomized, controlled group cognitive behavioural therapy for Internet addiction in adolescent students in Shanghai [J]. Australian & New Zealand Journal of Psychiatry, 2010, 44(2): 129-34.

[12] CHEN K, ZHENG W, MU S. Evaluation on the effect of group psychotherapy for Internet addiction students in Wenzhou Universities [J]. Chinese Journal of School Health, 2010, 31(06): 665-6.

[13] HUANG Z, QIAN M, ZHU S, et al. effects of interpersonal group counselling on college students with computer gaming addiction [J]. Chinese Mental Health Journal, 2010, 24(01): 29-33.

[14] LIU W, WEN B, YAO G, et al. efficacy of centralized and closed group counseling on internet-dependent undergraduates [J]. Chinese Journal of School Health, 2010, 31(04): 390-1+4.

[15] SU W, FANG X, MILLER J K, et al. Internet-based intervention for the treatment of online addiction for college students in China: a pilot study of the Healthy Online Self-helping Center [J]. Cyberpsychology, behavior and social networking, 2011, 14(9): 497-503.

[16] CHEN Z, TANG Y, ZHANG X, et al. Evaluation of intervention effect of staged group counseling on Internet addicted College Students [J]. Chinese Journal of School Health, 2011, 32(06): 726-8.

[17] QIU Q. experimental study of e-sprts' effects on addictive behaviors [J]. journal of shenyang sports university, 2011, 30(2).

[18] KONG M, FU W, LIU W, et al. Effect of Group Guidance on Internet Addiction Disorder among University Students [J]. Chinese Journal of Clinical Psychology, 2011, 19(1).

[19] WANG Y. Follow-upstudyofcognitive-behaviortherapyon adolescentwith internetaddiction disorder [J]. Chinese journal of behavioral medicine and brain science, 2011, 20(3).

[20] MA N, HE C. case-control study of cognitive-behavior therapy in the tendency in the tendency of internet addiction and loneliness among the undergraduates [J]. Chinese journal of disease control and prevention, 2011, 15(6).

[21] DUAN S, YE M. "Psychological intervention in college students’ internet dependence and its effect:

Based on the control test in Hunan Police Academy" [J]. Journal of Hunan Agricultural University (Social Sciences), 2012, 13(06): 63-7.

[22] GAO J, SUN J, XIAO K. Influence of Sport Interference on University Students’Network Addiction [J]. Journal of Shenyang Sport University, 2012, 31(4): 55-9.

[23] GUAN M, QIAN S, LIU W. Effect of Group Therapy on Higher Vocational School Students with Internet Dependence [J]. Chinese General Practice, 2012, 15(34): 4000-2.

[24] LV W, ZHANG B. The Eff ects of Group Counsel ing on Internet- Dependent Col lege Students [J]. China Journal of Health Psychology, 2012, 20(12): 1845-7.

[25] WANG Q. Evaluation of intervention effect of internet addiction by cognitive behavioral therapy for college students [J]. journal of huangshi institute of technology, 2012, 28(4).

[26] XU W, LIU Z. A study on the intervention of group counseling based on self-control to middle school students' Internet Addiction [J]. Journal of Shijiazhuang University, 2012, 14(06): 92-5.

[27] LIU D, LU N, HE J, et al. Effects of internet addiction group counseling on internet using and study management in college students [J]. Chinese Mental Health Journal, 2013, 27(7): 496-501.

[28] LU Y, YANG Y, MU J, et al. The influence of Satir family therapy on family cohesion and family adaptability in internet addictive disorder [J]. Chinese Journal of Behavioral Medicine and Brain Science, 2013, 22(5): 425-7.

[29] ZHANG C. "A Study on the Sports Intervention of Undergraduates’ Internet Addiction in

Kunming City" [J]. SPORT SCIENCE AND TECHNOLOGY, 2013, (4).

[30] DENG S, LI L, LIU J. Effects of integrated behavior intervention on internet addictive disorder among children [J]. Chinese Journal of Public Health, 2014, 30(07): 878-81.

[31] GE Y, HU Y, ZHANG Z, et al. "Effects of psychodrama group counseling on internet addiction and social

avoidance in urban left-behind children" [J]. Chinese Mental Health Journal, 2014, 28(06): 458-65.

[32] LI M, LI L, CHEN Y, et al. Current situation and exercise prescription intervention of Internet addiction among middle school students [J]. Chinese Journal of School Health, 2014, 35(12): 1847-8+52.

[33] LIU H. Research on group intervention of network dependence of primary and middle school students [J]. FUJIAN INSTITUTE OF EDUCATION, 2014, 15(02): 10-5+91+128.

[34] REN J, AO Z, HE C, et al. Experimental study on sports intervention of College Students' Internet Addiction [J]. HEILONGJIANG MEDICINE AND PHARMACY, 2014, 37(4): 93-4.

[35] WEI Q, YANG Y, WANG C, et al. an intervention program for both adolescnets with internet addiction disorder and their parents [J]. journal of nursing science 2014, 29(19): 68-71.

[36] LIU Q X, FANG X Y, YAN N, et al. Multi-family group therapy for adolescent Internet addiction: exploring the underlying mechanisms [J]. Addictive behaviors, 2015, 42: 1-8.

[37] CHENG Z, WANG Y, LI G. Effect of hypnosis combined with cognitive behavioral therapy on Internet addiction behavior of Internet addicted College Students [J]. Chongqing Medicine, 2015, (27): 3874-6.

[38] LI L, CHEN Y, HU Y, et al. Evaluation of the intervention effect of high-intensity intermittent exercise and dietary intervention on obese female college students with internet addiction disorder [J]. Chinese Journal of School Health, 2015, 36(10): 1466-8.

[39] SU X. Effects of group counseling on Internet addiction among vocational medical students [J]. Chinese Journal of School Health, 2015, 36(8).

[40] PENG W, LI Z. The Effect of group psychotherapy on internet addiction and mental health of secondary school students [J]. Journal of Clinical Psychosomatic Diseases, 2015, (4): 82-4.

[41] ZHAO H, HU X. An empirical study of College Students' Internet addiction on Group Counseling [J]. Ideological and Political Education Research, 2015, 31(01): 138-40.

[42] ÇELIK Ç B. Educational intervention for reducing Internet addiction tendencies [J]. The Turkish Journal on Addictions, 2016, 3(3): 375-86.

[43] LI L-L, SU Y, ZHEN G, et al. Effectiveness of a systematic nursing intervention model in adolescent patients with Internet addiction [J]. Chinese Journal of Modern Nursing, 2016, 22(23): 3357-60.

[44] FU Y, LIU Y. Analysis on the status of Internet Addiction of Korean college students and the effect of football training intervention [J]. Chinese Journal of School Health, 2016, 37(12): 1890-2.

[45] SU X. The intervention comparative study on the different dependence of vocational medical college students [J]. Chongqing Medicine, 2016, 45(24).

[46] ZHAO S, YANG Y, WEI Q, et al. Control study of curative effect on internet addiction disorder elimination by cognitive-be- havioral therapy in groups [J]. Medical Journal of the Chinese People Armed Police Forces, 2016, (4).

[47] KHAZAEI F, KHAZAEI O, GHANBARI-H B. Positive psychology interventions for internet addiction treatment [J]. Computers in human behavior, 2017, 72: 304‐11.

[48] YANG C-Y, ZENG G-F. The effect of tai chi exercise on college students' internet addiction [J]. Chinese Journal of School Health, 2017, 38(02): 292-4.

[49] LI L, HUANG Y. A study on the intervention of cognitive behavior group counseling on poor college students with Internet Addiction [J]. Modern Education Science, 2017, (04): 117-21.

[50] UYSAL G, BALCI S. Evaluation of a school-based program for Internet addiction of adolescents in Turkey [J]. Journal of addictions nursing, 2018, 29(1): 43-9.

[51] CHEN S, WU S. Effect of group cognitive behavioral therapy on adolescent Internet addiction patients [J]. Chinese and Foreign Medical Research, 2018, 16(15): 172-3.

[52] XU Z, XU H. Clinical effect of collective psychotherapy on Internet addicted adolescents [J]. China Journal of Clinical Rational Drug Use, 2018, 11(36): 173-4.

[53] ZHANG L, WANG Y, LI G, et al. Intervention effect of family sandplay therapy on adolescent internet addiction [J]. Journal of Guangdong Medical University, 2018, 36(3): 263-6.

[54] WöLFLING K, MüLLER K W, DREIER M, et al. Efficacy of Short-term Treatment of Internet and Computer Game Addiction: A Randomized Clinical Trial [J]. JAMA psychiatry, 2019, 76(10): 1018-25.

[55] FANG H, DAI Y. Psychological intervention of positice psychology on internet addiction of college students [J]. China journal of health psychology, 2019, 27(12): 1906-10.

[56] JEONG H, OH J K, CHOI E K, et al. Effects of transcranial direct current stimulation on addictive behavior and brain glucose metabolism in problematic online gamers [J]. Journal of behavioral addictions, 2020, 9(4): 1011-21.

[57] WEN L-J, CHEN Y-H. Effectiveness of short-term high-intensity exercise combined with nutritional intervention in female college students with internet addcition and ovesity [J]. Chinese Journal of School Health, 2020, 41(01): 51-4.

[58] ZHONG Y-H. Effect of rTMS on decreasing internet addicts' craving for internet [J]. Journal of gannan madical university, 2020, 40(07): 727-9.

[59] ALAVI S S, GHANIZADEH M, MOHAMMADI M R, et al. The effects of cognitive-behavioral group therapy for reducing symptoms of internet addiction disorder and promoting quality of life and mental health [J]. Trends in psychiatry and psychotherapy, 2021, 43(1): 47-56.

[60] LEE J Y, JANG J H, CHOI A R, et al. Neuromodulatory Effect of Transcranial Direct Current Stimulation on Resting-State EEG Activity in Internet Gaming Disorder: A Randomized, Double-Blind, Sham-Controlled Parallel Group Trial [J]. Cerebral cortex communications, 2021, 2(1): tgaa095.

[61] LIU X, JIANG J, ZHANG Y. Effects of Logotherapy-Based Mindfulness Intervention on Internet Addiction among Adolescents during the COVID-19 Pandemic [J]. Iranian journal of public health, 2021, 50(4): 789-97.

[62] LU Z, LI Z, YAN F. Effect of group cognitive therapy on negative emotion and impulsivity of college students with Internet addiction [J]. Chinese Journal of School Health, 2021, 42(6).

[63] GONG Y, GONG N, ZHANG Z, et al. Effect of Narrative Therapy-Based Group Psychological Counseling on Internet Addiction among Adolescents [J]. Iranian journal of public health, 2022, 51(6): 1331-8.

[64] LINDENBERG K, KINDT S, SZáSZ-JANOCHA C. Effectiveness of Cognitive Behavioral Therapy-Based Intervention in Preventing Gaming Disorder and Unspecified Internet Use Disorder in Adolescents: A Cluster Randomized Clinical Trial [J]. JAMA network open, 2022, 5(2): e2148995.

[65] YANG W, HU W, MORITA N, et al. Impact of Short-Term Intensive-Type Cognitive Behavioral Therapy Intervention on Internet Addiction among Chinese College Students: A Randomized Controlled Trial [J]. International journal of environmental research and public health, 2022, 19(9).

[66] ZHENG Y, HE J, FAN L, et al. Reduction of symptom after a combined behavioral intervention for reward sensitivity and rash impulsiveness in internet gaming disorder: A comparative study [J]. Journal of psychiatric research, 2022, 153: 159-66.
